# Alcohol milestones and internalizing, externalizing, and executive function: longitudinal and polygenic score associations

**DOI:** 10.1017/S003329172400076X

**Published:** 2024-05-09

**Authors:** Sarah E. Paul, David A.A. Baranger, Emma C. Johnson, Joshua J. Jackson, Aaron J. Gorelik, Alex P. Miller, Alexander S. Hatoum, Wesley K. Thompson, Michael Strube, Danielle M. Dick, Chella Kamarajan, John R. Kramer, Martin H. Plawecki, Grace Chan, Andrey P. Anokhin, David B. Chorlian, Sivan Kinreich, Jacquelyn L. Meyers, Bernice Porjesz, Howard J. Edenberg, Arpana Agrawal, Kathleen K. Bucholz, Ryan Bogdan

**Affiliations:** 1 Department of Psychological and Brain Sciences, Washington University in St. Louis, St. Louis, MO, USA;; 2 Department of Psychiatry, Washington University School of Medicine, St. Louis, MO, USA;; 3 Population Neuroscience and Genetics (PNG) Center, Laureate Institute for Brain Research, Tulsa, OK, USA;; 4 Department of Psychiatry, Robert Wood Johnson Medical School, Rutgers University, Piscataway, NJ, USA;; 5 Rutgers Addiction Research Center, Rutgers University, Piscataway, NJ, USA;; 6 Department of Psychiatry and Behavioral Sciences, SUNY Downstate Health Sciences University, Brooklyn, NY, USA;; 7 Department of Psychiatry, Carver College of Medicine, The University of Iowa, Iowa City, IA, USA;; 8 Department of Psychiatry, School of Medicine, Indiana University, Indianapolis, IN, USA;; 9 Department of Psychiatry, School of Medicine, University of Connecticut, Farmington, CT, USA;; 10 Department of Medical and Molecular Genetics, School of Medicine, Indiana University, Indianapolis, IN, USA; 11 Department of Biochemistry and Molecular Biology, School of Medicine, Indiana University, Indianapolis, IN, USA

**Keywords:** Alcohol initiation, alcohol intoxication, alcohol use disorder, internalizing, social anxiety, suicidal ideation, externalizing, ADHD, conduct disorder, executive function, polygenic scores, longitudinal

## Abstract

**Background.:**

Although the link between alcohol involvement and behavioral phenotypes (e.g. impulsivity, negative affect, executive function [EF]) is well-established, the directionality of these associations, specificity to stages of alcohol involvement, and extent of shared genetic liability remain unclear. We estimate longitudinal associations between transitions among alcohol milestones, behavioral phenotypes, and indices of genetic risk.

**Methods.:**

Data came from the Collaborative Study on the Genetics of Alcoholism (*n* = 3681; ages 11–36). Alcohol transitions (first: drink, intoxication, alcohol use disorder [AUD] symptom, AUD diagnosis), internalizing, and externalizing phenotypes came from the Semi-Structured Assessment for the Genetics of Alcoholism. EF was measured with the Tower of London and Visual Span Tasks. Polygenic scores (PGS) were computed for alcohol-related and behavioral phenotypes. Cox models estimated associations among PGS, behavior, and alcohol milestones.

**Results.:**

Externalizing phenotypes (e.g. conduct disorder symptoms) were associated with future initiation and drinking problems (hazard ratio (HR)⩾1.16). Internalizing (e.g. social anxiety) was associated with hazards for progression from first drink to severe AUD (HR⩾1.55). Initiation and AUD were associated with increased hazards for later depressive symptoms and suicidal ideation (HR⩾1.38), and initiation was associated with increased hazards for future conduct symptoms (HR = 1.60). EF was not associated with alcohol transitions. Drinks per week PGS was linked with increased hazards for alcohol transitions (HR⩾1.06). Problematic alcohol use PGS increased hazards for suicidal ideation (HR = 1.20).

**Conclusions.:**

Behavioral markers of addiction vulnerability precede and follow alcohol transitions, highlighting dynamic, bidirectional relationships between behavior and emerging addiction.

Excessive alcohol use is a major global health concern, typically beginning in adolescence and accounting for over 5% of global death and disability and 20% of deaths of those aged 20–49 in the U.S. (Esser et al., 2022; Forouzanfar et al., 2016). Alcohol use disorder (AUD) is among the most prevalent (29.1% lifetime) and comorbid psychopathologies (Castillo-Carniglia, Keyes, Hasin, & Cerdá, 2019). At the same time, few receive treatment (7.6%–19.8%; Grant et al., 2015; Olfson, Blanco, Wall, Liu, & Grant, 2019), and there are high relapse rates among those who do (Moos & Moos, 2006). Despite appreciable advances in AUD research (Koob & Volkow, 2016; Zhou et al., 2020), differentiation between the causes and consequences of AUD as it progresses is incomplete. Given the substantial burden of alcohol involvement, it is of critical public health interest to better understand the developmental course of alcohol use and AUD in relationship to genetic liability and behavioral precipitants and/or sequelae.

The neurobiological stage-based model of addiction postulates that alcohol-induced alterations in neural circuits promote three addiction stages—Binge/Intoxication, Withdrawal/Negative Affect, and Preoccupation/Anticipation (Koob & Volkow, 2010, 2016)— that broadly correspond with impulsivity/incentive salience, negative emotionality, and reduced executive function, respectively. Stimulation of neural reward circuits positively reinforces escalating use and potentiates impulsive behaviors (Binge/Intoxication). Following continued heavy use, alcohol intake becomes predominantly negatively reinforcing and compulsive, functioning to alleviate negative affect and withdrawal (Withdrawal/Negative Affect). Repeated alcohol-reward and alcohol-relief pairings beget craving and preoccupation with alcohol use (Preoccupation/Anticipation) and a loss of control over alcohol use arising from impaired prefrontal control of circuits that drive reward- and affect-related processes (Heather, 2020; Koob & Volkow, 2010, 2016).

Other models of addiction emphasize the predispositional role of traits aligned with these addiction stages (i.e. impulsivity, negative emotionality, and EF) in the initial use of alcohol and development of problems. The neurodevelopmental model theorizes that typical patterns of uneven brain development during adolescence and young adulthood drive developmentally normative increases in impulsivity, negative affect, and urgency, as well as reduced executive control, that confer vulnerability to the positive and negative reinforcing properties of alcohol and to preoccupation (Casey, Heller, Gee, & Cohen, 2019; Casey & Jones, 2010; Heller, Cohen, Dreyfuss, & Casey, 2016; Rapuano, Rosenberg, & Maza, 2020). Broadly, models of AUD suggest that trait-like differences in impulsivity, negative affect, and EF may indicate predispositional risk for AUD development and/or consequences of alcohol exposure (Bogdan, Hatoum, Johnson, & Agrawal, 2023; Kramer et al., 2020; Kwako, Momenan, Litten, Koob, & Goldman, 2016; Littlefield, Stevens, & Sher, 2014; Rawls, Kummerfeld, & Zilverstand, 2021). However, few studies have evaluated longitudinal bidirectional relationships between AUD stages and these ‘stage-based’ behavioral indicators.

## Bidirectional associations between behavior and alcohol involvement

Extensive longitudinal research shows that elevated impulsivity and related clinical phenotypes (e.g. ADHD) prospectively predict alcohol involvement (e.g. initiation, consumption, problematic use; Chassin, Pitts, & Prost, 2002; Defoe, Khurana, Betancourt, Hurt, & Romer, 2022; Elkins, King, McGue, & Iacono, 2006; Farmer et al., 2016; Fergusson, Horwood, & Ridder, 2007; King, Iacono, & McGue, 2004; Kuperman et al., 2013; Quinn & Harden, 2013; Stautz & Cooper, 2013; Wright & Jackson, 2023), in line with neurodevelopmental theories that impulsivity poses vulnerability to the initial seeking and rewarding aspects of alcohol use. A smaller body of work in large, prospective samples of youth and young adults suggests that alcohol use may be linked with increases in impulsive and externalizing phenotypes, possibly reflecting alcohol-induced neurocognitive impairment, reward learning, and/or shared genetic predisposition, consistent with the neurobiological stage-based model (Quinn, Stappenbeck, & Fromme, 2011; Staff, Maggs, Bucci, & Mongilio, 2019).

The relationship between internalizing phenotypes and alcohol involvement is more nuanced. With few exceptions (Ning, Gondek, Patalay, & Ploubidis, 2020), internalizing has not been strongly linked to early alcohol involvement (e.g. initiation, intoxication) but has been prospectively linked to more severe alcohol engagement (e.g. AUD, relapse), with evidence that alcohol involvement may also potentiate internalizing symptoms (Bucholz et al., 2017; Elkins et al., 2006; Farmer et al., 2016; Flórez-Salamanca et al., 2013; King et al., 2020; McCabe, Brumback, Brown, & Meruelo, 2023; Menary, Corbin, & Chassin, 2017). Interestingly, some evidence suggests that internalizing symptoms and behavioral inhibition may initially protect against alcohol use in early adolescence (e.g. via reduced social use opportunities), but then confer risk for more rapid progression of use later when more opportunities to drink arise (Hussong, Ennett, Cox, & Haroon, 2017; Ning et al., 2020).

Deficits in executive function (EF) often act as premorbid vulnerability factors that may promote initial alcohol-seeking and problematic use, and worsen alongside escalating severity of AUD, as proposed in the neurobiological theory. For example, EF deficits (e.g. in response inhibition) may hamper regulation of impulsive urges to engage in risky drinking (Jones, Meier, Corbin, & Chassin, 2021). In a large longitudinal study of adolescents, associations between alcohol use and impairments in working memory and inhibitory control were attributable to common vulnerability rather than neurotoxicity (Morin et al., 2019). Although performance in some domains of cognition shows evidence of recovery following abstinence, impairments in working memory and inhibitory control appear to persist (Nowakowska-Domagała, Jabłkowska-Górecka, Mokros, Koprowicz, & Pietras, 2017; Stavro, Pelletier, & Potvin, 2013; but see Pitel et al., 2009). It may be that executive dysfunction confers vulnerability to escalating use following initiation and worsens in some domains as use becomes chronic and problematic.

In sum, prominent theories of AUD development endeavor to explain its associations with impulsivity, negative affect, and EF. Existing longitudinal studies provide some evidence of bidirectional associations, with some specificity (e.g. internalizing more strongly linked to later milestones). Longitudinal studies that not only measure these traits both before and after relevant alcohol milestones but also incorporate indicators of genetic liability can be further used to parse the directionality and potential shared etiology of these relationships, especially when considering potentially censored observations (e.g. later expression of internalizing symptoms, limited phenotypic expression of high genetic risk).

## Genetic contributions to associations between behavioral phenotypes and alcohol involvement

There is robust support for heritable influences on alcohol use and AUD (twin heritability ~30–40% and 50%, respectively; Koopmans, Slutske, van Baal, & Boomsma, 1999; Verhulst, Neale, & Kendler, 2015; Viken, Kaprio, Koskenvuo, & Rose, 1999), as well as for impulsivity, risk-taking, negative urgency, depression, and EF (Anokhin, Grant, Mulligan, & Heath, 2015; Flint & Kendler, 2014; Friedman et al., 2008; Gustavson et al., 2020), with well-powered genome-wide association studies (GWAS) identifying loci that contribute to this heritable variation (Hatoum et al., 2023; Karlsson Linnér et al., 2019; Levey et al., 2021; Zhou et al., 2020). Incorporating estimates of this genetic liability into theoretical frameworks and analytic models of the relationships between these phenotypes can help clarify the extent to which their association is explained by shared genetic predisposition. Indeed, the Genetically Informed Neurobiological model of Addiction (GINA; Bogdan et al., 2023) posits that genetically influenced individual differences in impulsivity/risk-taking, negative urgency and depression, and EF confer differential vulnerability to the initial seeking and acutely rewarding aspects of substances, heightened negative affect with chronic use, and difficulty engaging in goal-directed behavior when salient substance-related cues elicit craving.

There is some evidence that genetic correlations and associations with polygenic scores (PGS) show stronger genetic relationships between externalizing and alcohol initiation/consumption and between internalizing and problematic use. Problematic alcohol use, but not consumption, is genetically correlated with indices of psychopathology (Sanchez-Roige et al., 2019; Walters et al., 2018; Zhou et al., 2020). Evidence further shows opposing genetic correlations in which alcohol frequency and quantity are associated with reduced and increased risk for psychopathology, respectively (Mallard et al., 2022; Marees et al., 2020; Sanchez-Roige et al., 2019). PGS for risk-taking is associated with future alcohol consumption (Ksinan, Su, Aliev, Spit for Science Workgroup, & Dick, 2019), while PGS for major depression is related to problematic use and dependence (Andersen et al., 2017; Casey et al., 2019). Despite the currently limited clinical utility of PGS (Rodrigue et al., 2023), investigating whether patterns of association between behavioral stage-based PGS and alcohol milestones, and, reciprocally, between alcohol PGS and stage-based symptom onsets, mirror phenotypic associations would yield important insight into the nature of these relationships. For instance, identifying PGS associations in the context of no symptoms or subthreshold symptoms poorly captured by categorical assessment would highlight the plausibility of shared genetic liability that may not yet be phenotypically expressed at the level of measurement.

## Current study

We assessed whether non-substance psychiatric representations of stage-based addiction phenotypes confer risk for progression of alcohol milestones and/or arise following these milestones. We leverage the Collaborative Study on the Genetics of Alcoholism (COGA) Prospective Study, a large (*n* = 3681), deeply pheno-typed, and prospectively assessed cohort of individuals aged 12–21 at wave 1 to examine prospective and bidirectional associations between stage-based phenotypes (i.e. externalizing, internalizing, and EF) and progression across alcohol milestones (i.e. first drink, first intoxication, first AUD symptom(s), first AUD diagnosis; Bucholz et al., 2017). We use psychopathology symptom onsets as proxies for stage-based phenotypes for several reasons:

1. Patterns of comorbidity between AUD and externalizing and internalizing disorders parallel links between stages of alcohol involvement and the core constructs of impulsivity and negative affect, respectively, implicated in these disorders;
2. Retrospectively reported onsets of such symptoms are carefully defined in this cohort, whereas continuous measures of impulsivity and negative affect may not be assessed before alcohol milestones;
3. Whether distinct subtypes of internalizing and externalizing disorders are specifically and bidirectionally related to alcohol involvement in ways consistent with the stage-based model has not yet been comprehensively explored; and
4. Focusing on these clinical phenotypes facilitates translation to clinical practice and integration with genetic evidence of shared liability revealed from GWAS of clinical phenotypes.

Consistent with the last point, we also examine whether PGS of psychiatric stage-based phenotypes are related to specific alcohol milestones and whether PGS of alcohol consumption and problematic use are differentially related to these stage-based phenotypes. We hypothesize that externalizing phenotypes will be broadly and reciprocally associated with all alcohol milestones, and that internalizing and EF will be primarily bidirectionally associated with later/more severe alcohol stages.

## Methods

### Sample

The Collaborative Study on the Genetics of Alcoholism (COGA) is an ongoing multi-site, family-based study designed to identify genes and traits associated with AUD (total *N*~17 762; Dick et al., 2023; Edenberg, 2002; Johnson et al., 2023; Reich et al., 1998). The COGA Prospective (COGA-P) Study (*n* = 3715) is a longitudinal sub-study of adolescent and young adult offspring with at least one parent who participated in the original COGA study and provided permission for offspring participation (Bucholz et al., 2017). All adult participants provided written informed consent, and minors verbal assent, to a protocol approved by each site’s institutional review board. Following quality control (Supplemental Methods), our final analytic sample included up to 15 463 observations from 3681 participants from 2186 nuclear families in 910 extended family pedigrees (51.3% female, 63.9% white, 27.4% Black, mean ± s.d. age at wave 1 = 16.02 ± 3.31 years; Table 1). Of these, 1935 and 918 are of European and African-American ancestry, respectively.

### Measures

#### Substance involvement and psychiatric assessment

Alcohol transitions and psychopathology onsets were derived from the Semi-Structured Assessment for the Genetics of Alcoholism interview (SSAGA; Bucholz, Cadoret, & Cloninger, 1994), which was administered approximately every 2 years (4.19 ± 2.07 times over 7.36 ± 4.40 years) to assess alcohol and substance use, problems, and disorders, as well as other psychiatric disorders (Table 2).

##### Alcohol Milestones/Transitions.

Alcohol milestones were defined as the age of the first: whole drink, intoxication, DSM-5 AUD symptom(s), and DSM-5 AUD diagnosis (Supplemental Methods). Consistent with prior COGA-P work (Bucholz et al., 2017; McCutcheon et al., 2018), transitions between these milestones (dichotomously coded 0/1 at each year within-person) were defined as follows: age 0 to first drink, first drink to first intoxication, first drink to first AUD symptom, and first drink to first AUD diagnosis (Table 2).

Given that age of first intoxication may be a better predictor of mental health outcomes (Newton-Howes, Cook, Martin, Foulds, & Boden, 2019), supplemental analyses defined the origin as first intoxication when predicting first AUD symptom and diagnosis (Supplemental Methods).

##### Externalizing and Internalizing.

The SSAGA inquires about ages of the first experienced impairing symptoms for each disorder examined. *Externalizing* (EXT) phenotypes were defined as the onset of: ADHD symptoms before age 12 (i.e. inattentive, hyperactive), persistent Oppositional Defiant Disorder symptoms, and ⩾3 Conduct Disorder symptoms. ***Internalizing*** (INT) phenotypes included the onsets of: a Major Depressive Episode, distressing Social Anxiety symptoms, Panic Attack accompanied by ⩾4 symptoms, core agoraphobia fear accompanied by additional agoraphobia symptoms, and suicidal ideation (Supplemental Methods). All EXT and INT phenotypes were dichotomous variables coded as 0/1 at each year, with ‘1’ indicating the onset of symptoms.

#### Executive function

The Tower of London Test (Shallice, 1982) and Visual Span Test (Milner, 1971) were used to assess executive function (i.e. planning and problem solving and visuospatial short-term memory, respectively; Supplemental Methods). *Tower of London* variables included: total excess moves made (errors), average pickup time (initial planning time), and average total time (total planning time). *Visual Span Test* variables included: highest sequences attained and total correct going forward and backward. For analyses predicting alcohol transitions, EF data came from the neuropsychological assessment conducted most recently prior to the alcohol transition (Supplement). No other neuropsychologically based cognitive assessments were collected in COGA-P.

#### Polygenic scores (PGS)

Details regarding the genomic data processing pipeline are provided in Supplemental Methods and in (Johnson et al., 2023; Lai, Wetherill, & Bertelsen, 2019). PGS were computed in the COGA-P European ancestry (EA) subsample (*n* = 1935) using PRS-CS (Ge, Chen, Ni, Feng, & Smoller, 2019), and in the African-American ancestry (AFR) subsample (*n* = 918) using PRS-CSx (Ruan et al., 2022; Supplemental Methods).

Ancestry-specific discovery GWAS were used to generate PGS for the following phenotypes (Table 3): *alcohol phenotypes* (i.e. Drinks/Week (Saunders et al., 2022), Problematic Alcohol Use (Kranzler et al., 2019; Zhou et al., 2020)), *internalizing* (i.e. major depressive disorder (Howard et al., 2019; Levey et al., 2021), generalized anxiety disorder (Levey et al., 2020), neuroticism (Nagel et al., 2018)), *externalizing* (i.e. risk tolerance (Karlsson Linnér et al., 2019), ADHD (Demontis et al., 2019), common executive function (Hatoum et al., 2023)). Because the present study examines stage-based behavioral phenotypes in relation to alcohol milestones, we do not use the recent, large externalizing GWAS (Karlsson Linnér et al., 2021) that includes alcohol and substance-related indicators. GWAS were selected based on sample size, relevance to phenotypes implicated in the neurobiological stage-based model, and similarity to the COGA-P phenotypes where possible (Supplemental Methods).

#### Covariates

All models (*statistical analysis*) covaried for: reported biological sex, highest parent educational attainment, parent household income, parent separation between the ages of 12 and 17, AUD case/control family status, parent AUD, birth cohort, and study site (Table 1), following prior work in this cohort (Bucholz et al., 2017). The following analysis-specific covariates were included in relevant models: White, Black, and those endorsing neither (self-reported) and Hispanic/not Hispanic (self-reported) in non-genetic analyses (Table 1); cigarette and cannabis use initiation and dependence in non-genetic analyses; first 10 ancestral principal components in genetic analyses; age of first drink and first intoxication in models analyzing survival from these milestones to adjust for any variability in risk period (Bucholz et al., 2017; Supplemental Methods).

### Statistical analysis

Cox proportional hazards models with mixed effects (Cox, 1972; Therneau, 2022) were used to generate hazard ratios (HR) for each predictor/outcome combination while including fixed effect covariates (above) and nuclear family as a random intercept to account for data non-independence. Models were run predicting: alcohol transitions from earlier EXT, INT, and EF phenotypes; EXT and INT symptom onsets from earlier alcohol milestones; alcohol transitions from EXT, INT, and EF PGS; and EXT and INT onsets from PGS for drinks/week and problematic alcohol use. Hazard ratio estimates required for 80% power ranged from 1.16 to 1.93 given sample sizes for models estimating survival to later stage-based symptom onsets excluding ADHD symptoms (see online Supplementary Methods; Supplementary Tables S14–S15). Due to insufficient numbers of events following alcohol milestones for agoraphobia and ADHD symptoms, these outcomes were excluded from analyses. Proportional hazards violations were investigated and resolved (Supplemental Methods).

Relationships between alcohol milestones and continuously assessed executive function (EF) development were assessed with linear mixed effects models (lme4 R package; Bates, Mächler, Bolker, & Walker, 2015). Time-in-study was person-mean-centered and used to model linear and quadratic within-person trajectories of EF. Main effects of alcohol milestones (Supplemental Methods) and interactions with time-in-study were explored with respect to each EF dependent variable. Random slopes allowed for varying rates of (linear) change across individuals. Statistically significant omnibus tests for the overall interaction effects were followed by *post hoc* pairwise comparison tests. The same analytic process was repeated for PGS, with PGS of drinks per week and problematic alcohol use replacing the ‘milestone’ variable (Supplemental Methods).

#### Multiple testing correction

False discovery rate (FDR) correction (Benjamini & Hochberg, 1995) was used to adjust for multiple testing. *p* values were adjusted across all predictors within EXT, INT, and EF separately, and separately across all alcohol milestone predictors of stage-based outcomes. Similarly, FDR correction was used across all INT PGS, all EXT PGS, and the EF PGS in predicting alcohol transitions, and across all alcohol PGS in predicting stage-based symptom onsets. For models examining EF trajectories, FDR correction was employed for the omnibus tests for the main effect of milestone and milestone * age interactions (14 tests), and for the 14 omnibus tests for the main effect of alcohol PGS and PGS * age interactions.

#### Post hoc severity analyses

Given heterogeneity in AUD severity, *post hoc* analyses examined whether stage-based phenotypes were associated with hazards for the onset of mild (2–3 criteria), moderate (4–5 criteria), and/or severe (6 + criteria) AUD (Supplemental Methods).

#### Post hoc analyses stratified by ancestry

Given that PGS analyses were conducted within each ancestral group (due to poor portability of PGS prediction across ancestries; Martin et al., 2017), phenotypic analyses were repeated in each ancestry group to facilitate comparison across phenotypic and PGS-based models (Supplemental Methods).

## Results

### Phenotypic associations

#### Ext, INT, and EF predicting alcohol transitions

##### Externalizing.

**Figure 1.**
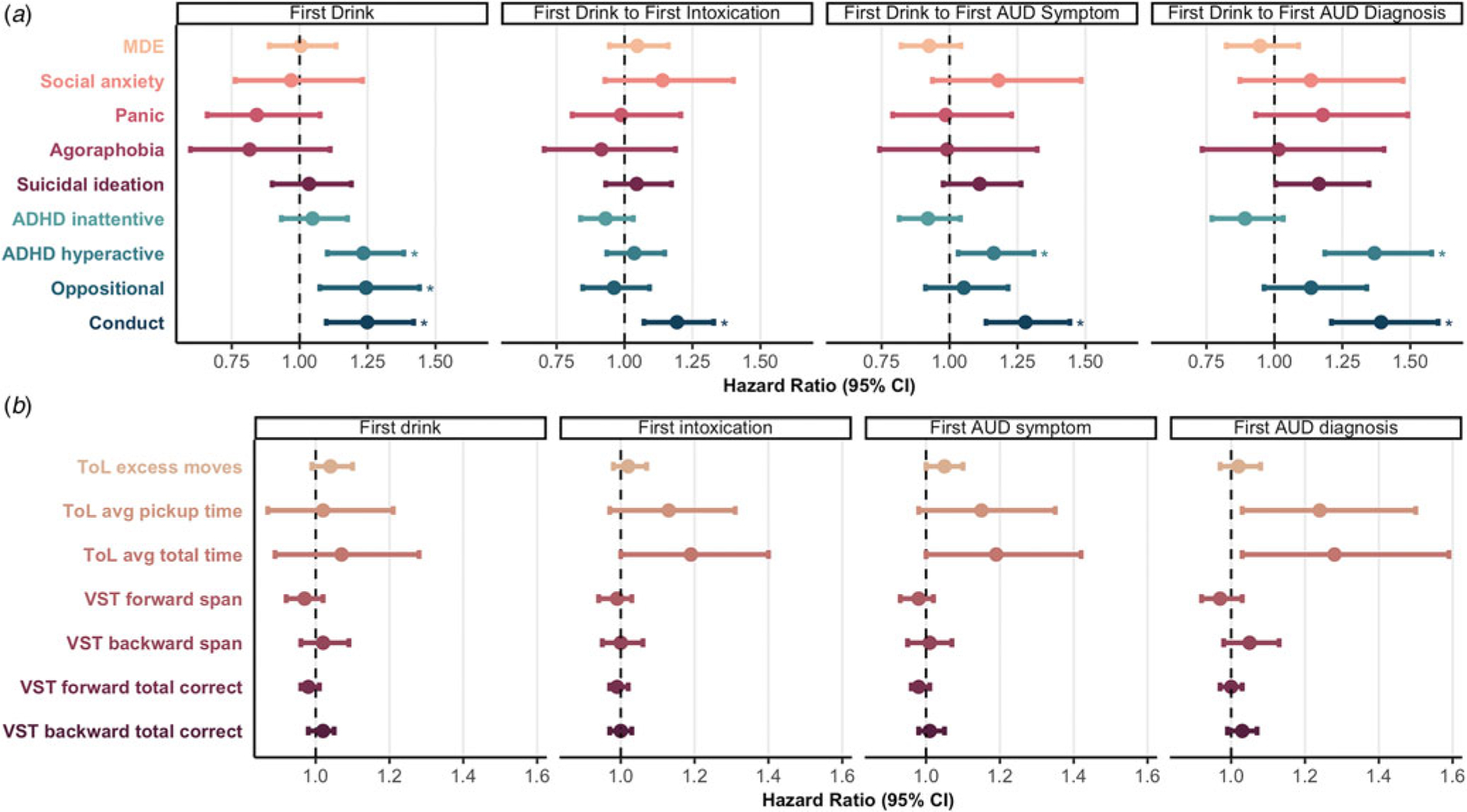
Associations between stage-based phenotypes and alcohol transitions. (a) Associations between internalizing and externalizing and hazards of alcohol transitions. Asterisks reflect estimates that survived FDR correction for all 20 tests for internalizing phenotypes, and separately, all 16 tests for externalizing phenotypes. Note three violations of the proportional hazards assumption for which time interactions were incorporated to resolve the violations: ADHD hyperactive symptoms in models of ‘First Drink’ and ‘First Drink to First Intoxication’ and ODD symptoms in the model of ‘First Drink to First Diagnosis.’ For simplicity, those interactions are not depicted here. All predictors were entered into the models simultaneously, alongside covariates. (b) Associations between executive function and hazards of alcohol transitions. ToL, Tower of London Test; VST, Visual Span Test. Note that for the ToL, higher scores reflect worse executive functioning, whereas the opposite is true for the VST. Hazard ratios for ToL measures are hazards associated with a doubling of the ToL measures, which were log2 transformed (Supplemental Methods). Each EF predictor was entered into analyses separately, given varying sample sizes and correlations among EF phenotypes. For both figures, point estimates reflect hazard ratios, and error bars represent 95% confidence intervals.

Conduct disorder symptoms were associated with increased hazards for all subsequent alcohol transitions (HR = 1.19–1.39, *p* ⩽ 1.30×10^−3^, *p*_FDR_ ⩽ 4.12×10^−3^; online Supplementary Table S1). ADHD hyperactive symptoms were also prospectively associated with increased hazards for all alcohol transitions (HR = 1.16–1.92, *p* ⩽ 0.014, *p*_FDR_ ⩽ 0.027; online Supplementary Table S1) except time from initiation to first intoxication (HR = 1.02–1.15, *p* ⩾ 0.19). ADHD inattentive symptoms were not significantly associated with hazards for any alcohol transition (0.89⩽HR⩽1.05, *p* ⩾ 0.13). Oppositional defiant disorder symptoms were associated with increased hazards for subsequent alcohol initiation transition to first AUD diagnosis (HR⩾1.24, *p* ⩽3.80 × 10^−3^, *p*_FDR_ = 9.03 × 10^−3^) but not other transitions (HR = 0.96–1.05, *p* ⩾ 0.18; Figure 1a; online Supplementary Table S1). *Post hoc* analyses of AUD severity showed that conduct disorder symptoms were linked with increased hazards of transitioning to moderate and severe AUD, hyperactive ADHD symptoms with transition to mild AUD, and oppositional defiant symptoms with transition to severe AUD (online Supplementary Fig. S1; Supplementary Table S2).

##### Internalizing.

INT was not significantly associated with hazards for any alcohol transitions (HR = 0.82–1.18, *p* ⩾ 0.043, *p*_FDR_ ⩾ 0.53; Figure 1a; online Supplementary Table S1). However, *post hoc* analyses revealed that social anxiety symptoms and suicidal ideation were significantly associated with increased hazards for progression from first drink to first severe AUD (online Supplementary Fig. 1; Supplementary Table S2).

##### Executive function.

EF was not significantly associated with any alcohol transition (HR = 0.97–1.28, *p* ⩾ 0.023, *p*_FDR_ ⩾ 0.32; Figure 1b, online Supplementary Table S3).

##### Post hoc analyses.

Supplementary analyses examining transitions from first intoxication to first AUD symptom and diagnosis were broadly consistent (online Supplementary Table S4).

#### Alcohol milestones predicting later EXT, INT, and EF

**Figure 2.**
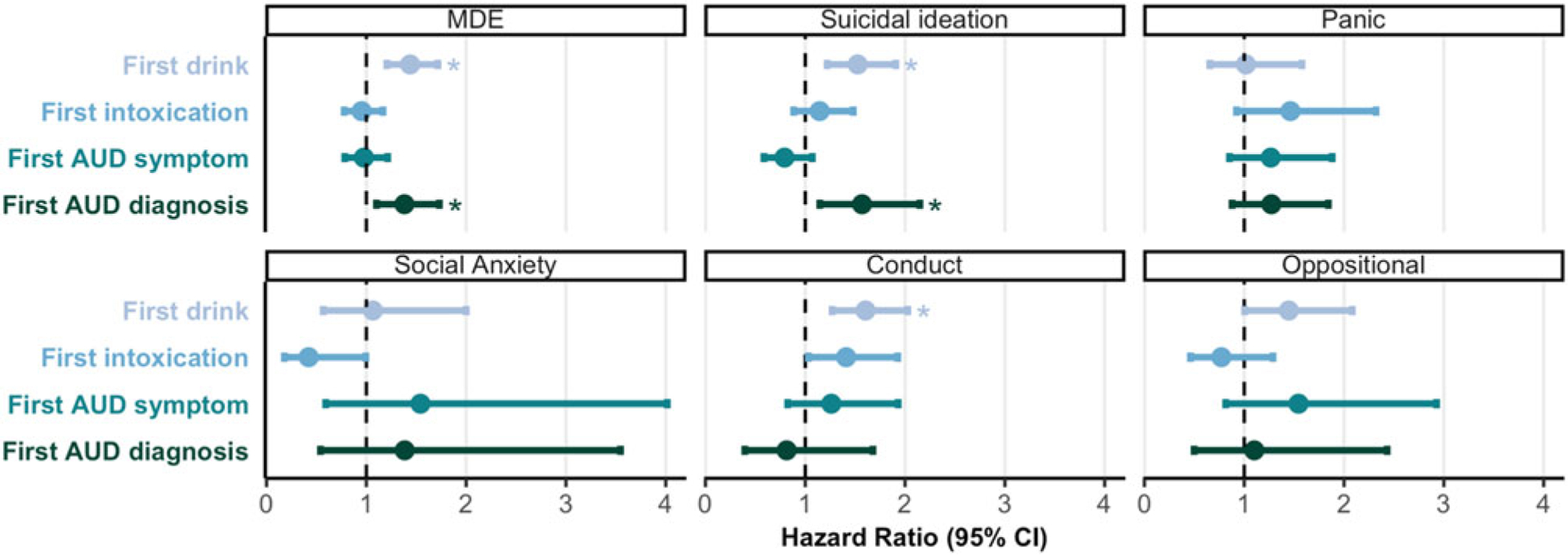
Associations between alcohol milestones and stage-based symptom onsets. Associations between alcohol milestones and hazards of stage-based symptom onsets. Point estimates reflect hazard ratios, and error bars represent 95% confidence intervals. Asterisks reflect estimates that survived FDR correction for all (24) tests. MDE, Major Depressive Episode; Panic, Panic Disorder Symptoms; Conduct, Conduct Disorder Symptoms; Oppositional, Oppositional Defiant Disorder Symptoms.

Alcohol initiation was significantly associated with increased hazards of subsequent onsets of INT and EXT (HR⩾1.44, *p* ⩽ 2.10×10^−4^, *p*_FDR_ ⩽ 1.68×10^−^3), except for panic, social anxiety, and oppositional defiant disorder symptoms (HR⩽1.45, *p* ⩾ 0.047, *p*_*FDR*_ ⩾ 0.147). Neither alcohol intoxication nor AUD symptom(s) was significantly associated with risk for any subsequent symptom onsets (0.42⩽HR⩽1.54, *p* ⩾ 0.031, *p*_*FDR*_ ⩾ 0.12). AUD diagnosis was associated with increased hazards of a subsequent major depressive episode and suicidal ideation (HR⩾1.38, *p* ⩽ 5.10×10^−3^, *p*_*FDR*_ = 0.024) but no other outcomes (HR = 0.81–1.38, *p* ⩾ 0.20; Figure 2; online Supplementary Table S5).

##### Executive function.

No significant omnibus effects were detected between alcohol milestones and EF trajectories (online Supplementary Table S6).

#### Genomic ancestry subsamples: phenotypic associations

Among both the European- and African-American Ancestry subsamples, phenotypic associations were broadly consistent with pattern seen in the full sample (Supplementary Tables S7–S8).

### Genetic associations

#### Stage-based phenotypes

**Figure 3.**
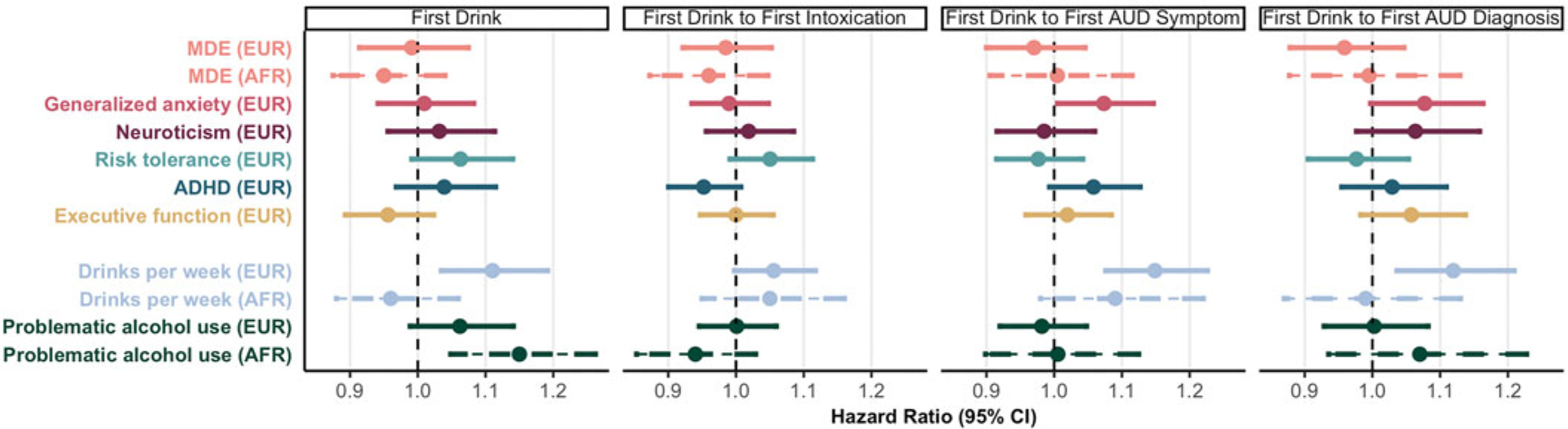
Associations between stage-based polygenic scores and alcohol transitions. Associations between stage-based PGS and hazards of alcohol transitions. EUR, PCA-selected European ancestry; AFR, PCA-selected African ancestry. Point estimates reflect hazard ratios, and error bars represent 95% confidence intervals. AFR ancestry PGS associations are depicted with dotted lines. Alcohol PGS are included for comparison at the bottom of each plot.

No PGS was significantly associated with alcohol transitions (HR = 0.95–1.08, *p* ⩾ 0.035, *p*_FDR_ ⩾ 0.29; Fig. 3; online Supplementary Table S9). Post hoc analyses showed that generalized anxiety disorder PGS was associated with faster progression from first intoxication to first AUD symptoms in the European ancestry subsample (HR = 1.10, *p* = 4.10 × 10^−3^, *p*_FDR_ = 0.025; online Supplementary Table S10).

*Post hoc* analyses of AUD severity showed that no PGS was significantly associated with hazards for AUD at any severity level in either ancestry (HR = 0.82–1.25, *p* ⩾ 6.60 × 10^−3^, *p*_FDR_ ⩾ 0.059; online Supplementary Fig. S2, Supplementary Table S11).

#### Alcohol involvement PGS

**Figure 4.**
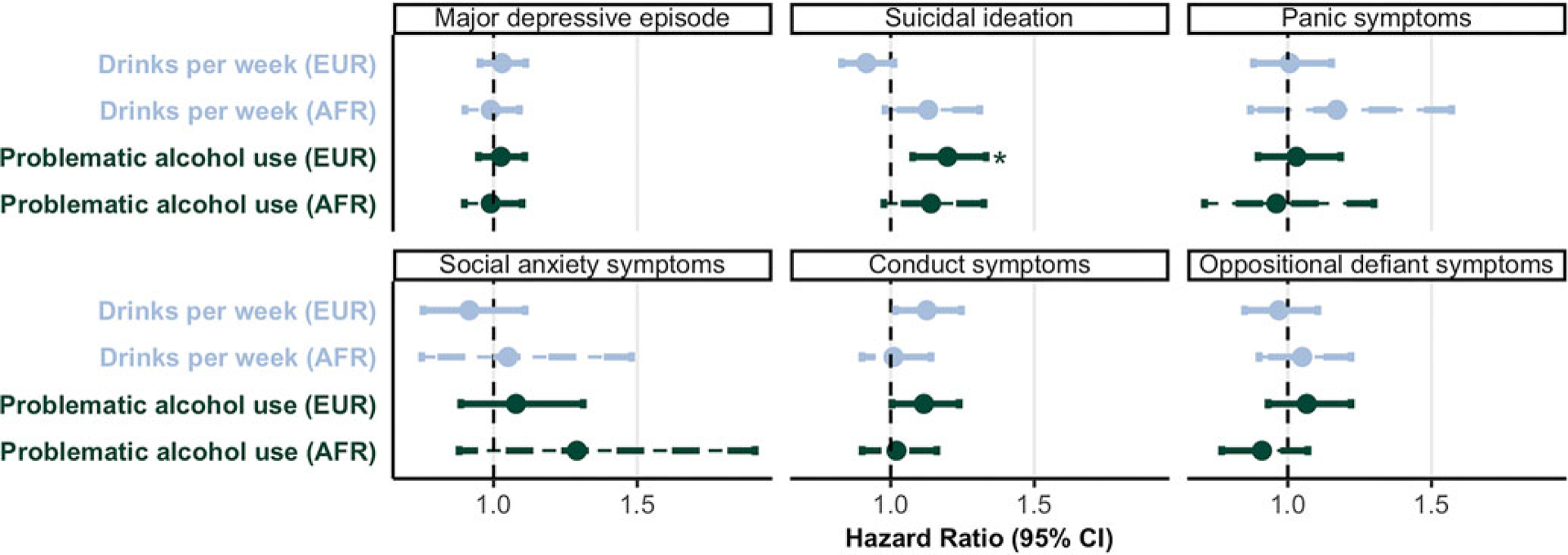
Associations between alcohol polygenic scores and stage-based outcomes. Associations between alcohol PGS and hazards of stage-based outcomes. Point estimates reflect hazard ratios, and error bars represent 95% confidence intervals. Solid lines represent PGS in EUR ancestry, and dashed lines represent PGS in AFR ancestry Asterisks reflect estimates that survived FDR correction for all 12 tests (2 alcohol PGS × 6 outcomes) in EUR ancestry. Note that, among individuals of AFR ancestry, models predicting panic symptoms, social anxiety symptoms, and oppositional defiant symptoms did not converge, so estimates may be unreliable.

Problematic Alcohol Use (PAU; Zhou et al., 2020) PGS was associated with increased hazards for suicidal ideation onset among individuals of European ancestry (HR = 1.20, *p* = 9.10 × 10^−4^, *p*_FDR_ = 0.011) but not with other phenotypes (HR = 0.94–1.29, *p* ⩾ 0.081). Drinks/week PGS showed no significant associations (HR = 0.92–1.18, *p* ⩾ 0.023, *p*_FDR_ ⩾ 0.14; Figure 4; online Supplementary Table S12). No significant omnibus effects were detected between alcohol involvement PGS and EF trajectories (online Supplementary Table S13). No significant omnibus effects were detected between alcohol involvement PGS and EF trajectories (online Supplementary Table S13).

## Discussion

Our study estimated longitudinal relationships between alcohol milestones/transitions (i.e. first drink, intoxication, AUD symptom, AUD diagnosis) and internalizing (INT), externalizing (EXT), and executive function (EF) phenotypes. By examining reciprocal time-varying associations, these findings critically extend the literature by clarifying the directionality and specificity of alcohol-psychopathology associations from early adolescence through young adulthood and the extent to which genetic liability maps onto phenotypic associations. Four broad findings emerged. *First,* EXT (i.e. ADHD hyperactive, oppositional defiant, and/or conduct disorder symptoms) was prospectively associated with increased hazards for all alcohol transitions. In contrast, only alcohol initiation prospectively predicted later EXT. *Second*, some INT phenotypes (i.e. social anxiety, suicidal ideation) were prospectively associated only with subsequent transitions to severe AUD. Alcohol milestones were prospectively associated with an accelerated risk for depression and suicidal ideation, but not panic or social anxiety symptoms. *Third*, EF (i.e. Tower of London Test, Visual Span Test) was not associated with hazards for any alcohol transition, nor were alcohol milestones associated with EF trajectories. *Fourth*, greater problematic alcohol use PGS was associated with increased hazards for suicidal ideation and the onset of conduct disorder symptoms; however, PGS for behavioral phenotypes were not significantly associated with alcohol transition hazards. Although the timing of typical developmental onsets and progressions of EXT, INT, and AUD may influence these longitudinal associations, these findings suggest that EXT symptomatology may promote accelerations across all stages of alcohol involvement while early INT may drive progression only to severe AUD and INT is more likely to arise following AUD.

### Externalizing symptoms are bidirectionally associated with alcohol milestones

As expected, EXT was robustly associated with all subsequent alcohol transitions, and earlier alcohol involvement was prospectively associated with increased hazards for EXT onset. Conduct disorder symptoms exhibited the most consistent associations with all alcohol transitions, and alcohol initiation increased hazards for conduct disorder symptoms. ADHD hyperactive—but not inattentive—symptoms were associated with all transitions except progression from initiation to intoxication and from intoxication to first AUD symptom. Oppositional defiant disorder symptoms were linked with increased hazards for future alcohol initiation, but not vice versa. These findings align with prior work showing that ‘disruptive behavior disorders” are associated with shorter time to alcohol problems and AUD onset (Edwards, Gardner, Hickman, & Kendler, 2016; Farmer et al., 2016), and that childhood/adolescent conduct symptoms and hyperactive—but not inattentive—ADHD symptoms are prospectively associated with later substance involvement (Elkins et al., 2018; Fergusson et al., 2007). Studies in young adults have documented associations between inattentive symptoms, which tend to be stable over time, and alcohol use and AUD (Glass & Flory, 2012; Grazioli et al., 2019; Roberts, Peters, Adams, Lynam, & Milich, 2014). Hyperactivity typically decreases with age (Biederman, Mick, & Faraone, 2000). In childhood, hyperactivity may show stronger associations with alcohol milestones/transitions, but in late adolescence or adulthood, inattention may appear more relevant. These findings suggest that externalizing is a more robust risk factor for alcohol initiation and escalation than the reverse, and that associations between alcohol milestones and subsequent EXT are restricted to early alcohol involvement. Of course, this pattern might be influenced by the typical development of EXT at earlier ages than problematic alcohol use (Kessler et al., 2005), or these associations might be due to shared underlying disposition that has variable expression across time (Hicks, Foster, Iacono, & McGue, 2013; Karlsson Linnér et al., 2021).

### Internalizing symptoms predict transitions to severe AUD, but not other alcohol transitions

Unlike associations with externalizing, internalizing symptoms were not associated with hazards for subsequent alcohol transitions. These results diverge from prior work in COGA-P showing that the presence of any internalizing disorder is associated with time to first AUD diagnosis (Bucholz et al., 2017). Sample sizes for certain individual disorders may have resulted in underpowered analyses, or perhaps an overarching internalizing domain confers more risk than specific disorders. However, post-hoc analyses painted a more nuanced picture; suicidal ideation and social anxiety were associated with increased hazards for progression from initiation to severe—but not mild or moderate—AUD. This pattern of results is consistent with recent analyses showing that AUD criteria mapping onto the Withdrawal/Negative Affect and Preoccupation/Anticipation stages are more frequently endorsed by those with severe AUD (Miller et al., 2023) and that anxiety and depression are linked with alcohol dependence symptoms more strongly than with initiation or quantity/frequency measures (Dick et al., 2014). Suicidal ideation has been cross-sectionally linked to more severe problematic alcohol use (Crossin et al., 2022), and suicide attempt has been longitudinally linked to future DSM-IV alcohol dependence in the COGA prospective cohort (Agrawal et al., 2017); present findings suggest that in a high-risk sample, suicidal ideation may escalate progression of AUD.

Social anxiety disorder symptoms were associated with a substantial increase in hazards for severe AUD, possibly attesting to vulnerability to high levels of alcohol-related problems once drinking is initiated and/or to strong negatively reinforcing effects of alcohol use on social anxiety. Prior work suggests that social anxiety may be uniquely, and possibly causally, related to AUD (Torvik et al., 2019), and that it is more strongly related to alcohol dependence or related problems than to DSM-IV abuse or consumption (Buckner et al., 2008; Schry & White, 2013).

### Alcohol milestones potentiate internalizing onsets

The accelerated development of depression and suicidal ideation following alcohol initiation and AUD onset, coupled with the absence of the reverse relationship, may be attributed to several factors. First, initial alcohol use and escalation may increase negative affect as alcohol use becomes more compulsive and non-alcohol rewards become less reinforcing, consistent with the stage-based model (Koob, 2015; Koob & Volkow, 2010, 2016). Prior work showed that the risk of depression onset following alcohol dependence is higher than the risk of alcohol dependence following depression (Fergusson, Boden, & Horwood, 2009; Gilman & Abraham, 2001; but see Polimanti et al., 2019). Second, internalizing may be unrelated to or protective against alcohol use, depending on developmental timing and/or levels of concurrent externalizing symptoms. Internalizing has been linked with future problematic alcohol use in adulthood but not adolescence (King et al., 2020), and internalizing symptoms may confer more risk at low levels of EXT (Colder et al., 2013, 2017). Third, early initiation and development of AUD may be linked with negative social, academic, and functional outcomes that increase risk for depression and suicidality (Miller, DiBello, Merrill, Neighbors, & Carey, 2020; Rosenthal et al., 2018). Fourth, gender differences may obscure relationships between internalizing and subsequent alcohol transitions, with prior work suggesting that early depressive symptoms may be associated with increased risk for later alcohol use and related harms more strongly in girls than boys (Edwards et al., 2014). Finally, we note that the onset of a major depressive episode in the SSAGA was not defined to be independent of drug or alcohol use, which is important to distinguish in future work (Schuckit, Smith, & Kalmijn, 2013).

### Polygenic scores for stage-based phenotypes are not associated with alcohol transitions

In contrast to phenotypic associations, and despite evidence for shared genetic liability to externalizing and AUD (Karlsson Linnér et al., 2021; Kendler, Prescott, Myers, & Neale, 2003), PGS for externalizing phenotypes were not significantly linked to hazards for alcohol transitions. GWAS of externalizing phenotypes (excluding substance-related variables) may be underpowered for the detection of cross-trait relationships with PGS (Carey, Agrawal, & Bucholz, 2016; Kember et al., 2021). Perhaps other stage-based phenotypes (e.g. negative urgency, inhibitory control), for which there are not yet GWAS, would exhibit more predictive utility.

### Executive function measures are not associated with alcohol milestones

Although impairments in executive function are thought to be a consequence of prolonged alcohol use and AUD (Koob & Volkow, 2010, 2016), the present study found that performance on two executive function tasks was unassociated with alcohol milestones as both predictor and outcome. Few studies have previously explored the relationship between alcohol consumption or AUD and performance on these tasks, with mixed findings (Hendriks, van de Rest, Snippe, Kieboom, & Hogenelst, 2020; Meyers et al., 2019; Pandey et al., 2018). Explanations for the present findings include the restriction of EF analyses to individuals with EF assessments that preceded milestone onsets (i.e. those that experienced milestones later, on average, may exhibit a lower constellation of risk factors) and the lack of measures of inhibitory control and other EF components more relevant to AUD.

## Strengths and limitations

The present study has notable strengths. Important alcohol transitions were examined, from first drink to the development of AUD. Alongside these transitions, behavioral indicators were examined both phenotypically and using PGS. Internalizing and externalizing phenotypes were measured by multiple discrete symptoms, allowing for fine-grained analysis that shows that social anxiety, suicidal ideation, conduct disorder, and hyperactive ADHD symptoms may be uniquely related to alcohol milestones. By examining bidirectional associations, a truly developmental model was adopted that begins to capture the dynamic process by which AUD and comorbid symptoms develop over time, with implications for intervention and etiologic understanding.

Some limitations are worth noting. *First*, although high-risk family-based samples have several benefits including enriched endorsement for AUD and other psychopathology, they may not generalize. *Second,* the PGS analyses were largely constrained to individuals of European ancestry due to the dearth of GWAS of these phenotypes in individuals of non-European ancestry and the poor portability of PGS prediction across ancestries (Martin et al., 2017). Despite our efforts to include PGS for those of African–American ancestry, the present null findings may be partially attributable to reduced power arising from smaller discovery GWASs in African ancestry populations. Our European ancestry-specific findings may thus contribute to the disparity in applicability of research findings to non-European populations (Martin et al., 2019). *Third,* ages of onsets of some symptoms for some participants were retrospectively reported and thus subject to recall bias, which we tried to minimize by choosing the first reported age of onset. *Fourth*, given insufficient assessments of dimensional psychopathology, we were unable to longitudinally evaluate subthreshold symptom development across waves, complicating the ability to distinguish predisposition from consequence. *Fifth*, despite covarying for tobacco and cannabis initiation and dependence, the identified pattern of associations may not be alcohol specific. *Sixth*, typical developmental timing of psychopathology onset (i.e. earlier onset of externalizing v. internalizing symptoms; Kessler et al., 2005) should be considered in the interpretation of findings; the prospective association between alcohol milestones and future internalizing may be partially attributable to low endorsement of internalizing prior to alcohol use onset, and the lack of significant associations between later alcohol milestones and future onsets of ODD and Conduct Disorder symptoms is likely due to generally earlier onset of EXT compared to later alcohol stages. *Seventh*, the age ceiling in the prospective study may have obscured relationships that are specific to individuals older than 36.

## Conclusions

In a sample of up to 3681 individuals aged 12–36, we found that externalizing psychopathology accelerates and is accelerated by alcohol transitions. Externalizing features (i.e. conduct disorder and hyperactive ADHD symptoms) were associated with all subsequent alcohol transitions, but only alcohol initiation increased hazards for externalizing. Internalizing features (i.e. suicidal ideation and social anxiety disorder symptoms) were only associated with increased hazards for severe AUD, with evidence that early (initiation) and later stages (AUD) may accelerate subsequent depression and suicidal ideation. We found no evidence that the EF measures used may modify the pace of alcohol transitions or vice versa.

The phenotypic link between AUD and future suicidal ideation was paralleled by the association between problematic alcohol use PGS and suicidal ideation, possibly suggesting that part of the phenotypic association is driven by shared genetic risk. Overall, the present study finds partial support for the notion that behavioral stage-based addiction phenotypes, as operationalized here, may not only reflect putative alcohol-related behavioral consequences but also developmental risk for AUD.

## Supplementary Material

Supplement

## Figures and Tables

**Table 1.** Participant characteristics

Variable	*n* (%)
Sex (% female)	1887 (51.3)
Race	
Black	1010 (27.4)
White	2351 (63.9)
Other^a^	320 (8.7)
Ethnicity (% Hispanic)	443 (12.0)
Highest parent education	
Less than high school	570 (15.6)
High school degree	895 (24.4)
Some college	1226 (33.5)
College degree	657 (17.9)
Graduate degree	317 (8.6)
Birth cohort	
1980–1986	842 (22.9)
1987–1989	786 (21.4)
1990–1993	954 (25.9)
1994+	1100 (29.9)
Parent household income	
<$30k	1289 (37.8)
$30k– $ 74 999k	1702 (49.9)
⩾$75k	418 (12.3)
Case family	3106 (84.4)
Parent AUD status	
No AUD or missing	1217 (33.1)
One possible AUD & one missing/no AUD	132 (3.6)
One AUD & one missing/no AUD	1512 (41.1)
Both AUD or one AUD & one possible AUD	818 (22.2)
Parent separation ages 12–17	1725 (46.9)
Study site	
Indiana University	458 (12.4)
University of Iowa	538 (14.6)
SUNY Downstate	573 (15.6)
University of Connecticut	746 (20.3)
University of California, San Diego	630 (17.1)
Washington University in St. Louis	736 (20.0)

AUD, alcohol use disorder.

a Native American, Asian, Pacific Islander, or ‘any other identified group.’.

*Note*: Characterization of COGA prospective study participants (total *n* = 3681).

**Table 2.** Participant SSAGA and executive function characteristics

Variable	*n* (%)	M (s.d.) Age onset
Alcohol initiation	3149 (85.6)	15.8 (2.6)
Alcohol intoxication	2840 (77.2)	16.8 (2.6)
Alcohol use disorder symptom	2153 (58.5)	17.7 (2.7)
Alcohol use disorder diagnosis	1506 (40.9)	18.6 (3.0)
Major depressive episode	1832 (49.8)	15.5 (4.3)
Social anxiety disorder	201 (5.5)	12.3 (4.6)
Panic disorder	397 (10.8)	16.5 (4.8)
Agoraphobia	175 (4.8)	15.0 (5.9)
Suicidal ideation	1106 (30.0)	15.2 (3.8)
ADHD inattentive	1503 (40.8)	6.8 (1.7)
ADHD hyperactive	1479 (40.2)	7.2 (1.6)
Oppositional defiant disorder	634 (17.2)	11.4 (3.2)
Conduct disorder	1128 (30.6)	12.8 (2.2)
Cigarette use	1791 (48.7)	15.8 (3.0)
Marijuana use	2537 (68.9)	16.2 (2.9)
Tobacco dependence	791 (21.5)	18.0 (2.9)
Marijuana dependence	741 (20.1)	17.4 (2.7)
Variable^a^	*n*	M (s.d.)
ToL excess moves made	2909	15.2 (14.3)
Tol average pickup time	2909	2.4 (0.84)
ToL average total time	2909	4.1 (1.3)
VST forward Span	2911	5.9 (1.4)
VST backward Span	2777	5.1 (1.1)
VST forward total correct	2911	8.4 (2.6)
VST backward total correct	2777	7.1 (2.1)

SSAGA, semi-structured assessment for the genetics of alcoholism. Major depressive episode, age most severe MDE OR first MDE; social anxiety disorder, first time symptoms made the individual upset with themselves; panic disorder, first time panic attack with 4 + symptoms; agoraphobia, first time fear with several other problems; suicidal ideation, first time had thoughts of taking own life; ADHD inattentive, first time 5 + ADHD inattentive symptoms with functional impairment; ADHD hyperactive, first time 5 + ADHD hyperactive symptoms with functional impairment; oppositional defiant disorder, first period of 6 + months with several symptoms; conduct disorder, first age at which individual experienced 3 + conduct disorder symptoms; cigarette use, first full cigarette; marijuana use, first marijuana use; ToL, Tower of London Test; VST, Visual Span Test.

a These *n*’s and descriptive statistics are for executive function measured at wave 1 (i.e. baseline wave).

*Note*: Characterization of stage-based and alcohol phenotypes. All numbers reflect lifetime status within the study timespan.

**Table 3.** Description of Genome-wide association studies (GWAS) used for polygenic score computation

Phenotype	*N*	$h_{\text{SNP}}^{2}$	Citation
**European ancestry**			
Drinks per Week (DPW)	666 978	0.040	Saunders et al. (2022)
Problematic Alcohol Use (PAU)	431 117^a^	0.068	Zhou et al. (2020)
Major Depressive Disorder (MDD)	570 414 (254 656 cases)	0.089	Howard et al. (2019)
	59 600 (25 843 cases)	0.113	Levey et al. (2021)
Generalized Anxiety Disorder (GAD)	175 163	0.056	Levey et al. (2020)
Neuroticism	390 278	0.100	Nagel et al. (2018)
Risk tolerance	466 571	0.046	Karlsson Linnér et al. (2019)
Attention-Deficit/Hyperactivity Disorder (ADHD)	55 374 (20 183 cases)	0.216	Demontis et al. (2019)
Executive function	427 037	0.104	Hatoum et al. (2023)
**African ancestry**			
Drinks per Week (DPW)	AA *N* = 8078 EA *N* = 666 978	0.049	Saunders et al. (2022)
Problematic Alcohol Use (PAU)	AA *N* = 56 468 EA *N* = 351 113	0.110	Kranzler et al. (2019)
Major Depressive Disorder (MDD)	AA *N* = 59 600 EA *N* = 750 414	0.113	Levey et al. (2021)

$h_{\text{SNP}}^{2}$, SNP-based heritability; AA, African Ancestry; EA, European Ancestry.

a Sample size excluding the COGA cohort from the discovery GWAS (*n* = 4386).

## Data Availability

COGA data are available through the National Institute on Alcohol Abuse and Alcoholism or the database of Genotypes and Phenotypes (dbGaP; phs000763.v1.p1, phs000125.v1.p1).
