# Supplementary material for: Alcohol milestones and internalizing, externalizing, and executive function: longitudinal and polygenic score associations
Source: Psychol Med. Author manuscript; Available in PMC 2024 Oct 10. (PMC11464200; doi:10.1017/S003329172400076X)
Supplement: Supplement [file NIHMS1996071-supplement-Supplement.docx]

**SUPPLEMENTARY MATERIALS**

**Longitudinal Relationships Between Alcohol Milestones and Internalizing, Externalizing, and Executive Function, and Associations with Polygenic Scores**

Sarah E. Paul, M.A.,^1^ David A.A. Baranger, Ph.D.,^1^ Emma C. Johnson, Ph.D.,^2^ Joshua J. Jackson, Ph.D.,^1^ Aaron J. Gorelik, M.A.,^1^ Alex P. Miller, PhD.,^2^ Alexander S. Hatoum, PhD.,^1^ Wesley K. Thompson, Ph.D.,^3^ Michael Strube, Ph.D.,^1^ Danielle M. Dick, Ph.D.,^4,5^ Chella Kamarajan, Ph.D.,^6^ John R. Kramer, Ph.D.,^7^ Martin H. Plawecki, MD, Ph.D.,^8^ Grace Chan, Ph.D.,^7,9^ Andrey P. Anokhin,^2^ David B. Chorlian, M.S., M.A.,^6^ Sivan Kinreich, Ph.D.,^6^ Jacquelyn L. Meyers, Ph.D.,^6^ Bernice Porjesz, Ph.D.,^6^ Howard J. Edenberg, Ph.D.,^10,11^Arpana Agrawal, Ph.D.,^2^ Kathleen K. Bucholz, Ph.D.,^2^ Ryan Bogdan, Ph.D.^1^

^1^ Department of Psychological and Brain Sciences, Washington University in St. Louis, St. Louis, MO USA

^2^ Department of Psychiatry, Washington University School of Medicine, St. Louis, MO USA

^3^ Laureate Institute for Brain Research, Tulsa, OK USA

^4^ Department of Psychiatry, Robert Wood Johnson Medical School, Rutgers University, Piscataway, NJ, USA

^5^ Rutgers Addiction Research Center, Rutgers University, Piscataway, NJ, USA

^6^ Department of Psychiatry and Behavioral Sciences, SUNY Downstate Health Sciences University, Brooklyn, NY USA

^7^ Department of Psychiatry, Carver College of Medicine, The University of Iowa, Iowa City, IA, USA

^8^ Department of Psychiatry, School of Medicine, Indiana University, Indianapolis, IN, USA.

^9^ Department of Psychiatry, School of Medicine, University of Connecticut, Farmington, CT, USA

^10^ Department of Medical and Molecular Genetics, School of Medicine, Indiana University, Indianapolis, IN, USA

^11^ Department of Biochemistry and Molecular Biology, School of Medicine, Indiana University, Indianapolis, IN, USA.

**Table of Contents**

**Supplemental Methods   3-9**

Measures     3-5

Analyses     4-8

Genotyping, Quality Control, and Polygenic Score (PGS) Computation    8-9

**Supplemental Results   10-11**

Transitions from first intoxication to first AUD symptom and first AUD diagnosis        10

Results stratified by PCA-selected ancestry group     10

Severity analyses       11

Executive function trajectories   11

Power Analysis 11

**Supplementary Tables and Figures     12-36**

Supplementary Table 1. Results for stage-based phenotypes and alcohol transitions       12

Supplementary Table 2. Results for stage-based phenotypes and AUD severity        14

Supplementary Table 3. Results for executive function and alcohol transitions        16

Supplementary Table 4. Results for stage-based phenotypes and alcohol         18

   transitions from first intoxication

Supplementary Table 5. Results for alcohol milestones and stage-based symptom          19

  onsets

Supplementary Table 6. Results for omnibus tests of alcohol milestone         20

   and age interactions in executive function trajectories

Supplementary Table 7. Results for stage-based phenotypes and alcohol transitions,      21

stratified by ancestry

Supplementary Table 8. Results for alcohol milestones and stage-based symptom          24

  onsets, stratified by ancestry

Supplementary Table 9. Results for PGS and alcohol transitions                               26

Supplementary Table 10. Results for PGS and alcohol transitions from first                   28

                 intoxication

Supplementary Table 11. Results for polygenic scores and AUD severity         29

Supplementary Table 12. Results for PGS and stage-based symptom onsets         31

Supplementary Table 13. Results for omnibus tests of alcohol PGS and age         32

    interactions in executive function trajectories

Supplementary Table 14. Power analyses for alcohol transitions         33

Supplementary Table 15. Power analyses for stage-based symptom onsets         34

Supplementary Figure 1. Results for stage-based phenotypes and AUD severity   35

Supplementary Figure 2. Results for stage-based PGS and AUD severity   36

**Supplemental References 37-42**

**Supplemental Methods**

**Measures**

**SSAGA Quality control.** Interviewers who administered the SSAGA rated participants on the “accuracy” of their reporting on a Likert-type scale ranging from 1 (“No difficulty”) to 4 (“Impossible to rate with any confidence”). Assessments that received an accuracy score of “4” were excluded from analyses, as were participants for whom >50% of their longitudinal assessments received accuracy scores of “3” (i.e., “Major difficulty in conducting exam”) or greater. A total of 34 individuals and 110 rows of data were excluded based on these criteria.

Although retrospective reporting may be prone to recall biases, the procedures to administer the SSAGA interview include steps to increase the accuracy of retrospective reports. The semi-structured nature of the SSAGA allows interviewers to ask for clarification if responses seem inconsistent or improbable; interviewers rate the overall reliability of the interview information, and we excluded highly unreliable interviews from the present analyses. The SSAGA uses a timeline for participants, aiming to map onsets to major life events; for participants with multiple reports of ages of onset; we used the first reported age of onset to minimize retrospective recall bias.

**Alcohol milestones.** First drink was defined as self-reported age (in years) at which an individual consumed their “very first whole drink,” described as, “a glass of wine, a can or bottle of beer, or a shot of liquor alone or in a mixed drink.” First intoxication was defined as the self-reported age at which an individual first “got drunk” or experienced slurred speech or unsteadiness. First AUD symptom was defined as the first self-reported age at which an individual experienced any symptom of AUD. First AUD diagnosis was defined as the first self-reported age at which an individual met diagnostic criteria for DSM-5 Alcohol Use Disorder.

Supplemental analyses (see **Analyses** below) examining time to first mild, moderate, and severe AUD diagnosis used the first self-reported age at which an individual met diagnostic criteria for mild (i.e., 2-3 symptoms), moderate (i.e., 4-5 symptoms), or severe (i.e., 6 or more symptoms) AUD.

**Externalizing.**

***ADHD symptoms.*** Participants were asked whether they experienced any of 10 symptoms related to inattention for a period of at least 6 months between the ages of 6 and 10, and those that reported at least 3 such symptoms along with any impairment were asked at what age they first experienced any of these symptoms. The first age reported was used to define the onset of ADHD Inattentive symptoms. The same process was repeated for ADHD Hyperactive symptoms, for which 11 symptoms were queried.

***Conduct disorder symptoms.*** In the SSAGA, ages of onset are obtained for each of 21 behaviors as well as for the first time an individual exhibited 3 such behaviors within a 6-month period. The present study used the first self-report of the latter to define the onset of conduct disorder symptoms. The 21 behaviors included the following in addition to those listed in the DSM-5: getting suspended from school; both staying out late at night without permission *and* sneaking out after bedtime; challenging adults by refusing to do what they ask; throwing temper tantrums; blaming others when at fault; cheating on schoolwork, exams, or games.

***Oppositional defiant disorder symptoms.*** The first self-reported age at which an individual who endorsed at least 4 ODD symptoms first exhibited several symptoms of ODD within a 6-month period.

**Internalizing.**

***Major depressive episode.*** Ages of onset of an individual’s most severe episode of depression (i.e., at least two weeks in which they experienced depressed mood or anhedonia) and, if different, the onset of their first episode of depression were collected, and the first-reported lowest of these two ages of onset defined an individual’s first major depressive episode.

***Social anxiety symptoms.*** Participants were asked whether they had ever experienced “strong and persistent” fears of any of six situations (e.g., speaking to authority figures, eating in public, using public restrooms, talking to unfamiliar people), whether this fear interfered in their lives, whether such situations lead to immediate anxiety and cause/or avoidance, whether the fear was “excessive,” and whether the individual has been “very upset” when themself for these fears. Those that endorsed any phobic situation and all of the remaining symptoms were asked about the first time any of these fears made them “upset with” themselves, and this age of onset was used to mark the onset of social anxiety symptoms.

***Panic attack among those with Panic Disorder.*** The age of the first self-reported panic attack among those that endorsed having had at least several attacks that involved 4 or more panic symptoms and began suddenly and got worse within 10 minutes.

***Agoraphobia symptoms.*** The first self-reported age at which an individual had a fear of situations in which they felt it would be difficult to leave easily and experienced “some other problems,” such as physical symptoms and avoidance or extreme anxiety.

***Suicidal ideation.*** The first self-reported age at which an individual experienced persistent (i.e., at least 7 days) suicidal ideation and had a plan to end their life.

**Executive Function**

***Tower of London Test (TOLT).*** The TOLT tests problem solving and planning [(Shallice, 1982)](https://www.zotero.org/google-docs/?dvfH34). Participants are shown a completed puzzle with colored beads on pegs and are asked to move another set of colored beads on pegs until it matches the completed puzzle in as few moves as possible. Trials of 3, 4, and 5 beads are administered via computer using Colorado Assessment Tests (CATs) software[(Davis & Keller, 2002)](https://www.zotero.org/google-docs/?kBASr9).

Primary outcome measures for the TOLT used in the present study were total excess moves made, average pickup time, and total trials time across all trials. These measures have been used in previous studies in COGA [(Meyers et al., 2019; Subbie-Saenz de Viteri et al., 2020)](https://www.zotero.org/google-docs/?xgxnBy) and other high risk samples [(e.g., Kamarajan et al., 2020)](https://www.zotero.org/google-docs/?aRe1eI), though different combinations of measures are used across the different studies. We selected three measures given high correlations among the “time” (i.e., r = 0.84-1) measures and desire to minimize multiple testing.

Because these three TOLT outcome measures were skewed (skewness = 2.18-2.50), all values were log-transformed prior to analysis. Given the presence of “0”s in the excess moves made measure, “1” was added to all values prior to log transformation.

***Visual Span Test (VST).*** The CATS-Visual Span test [(Milner, 1971)](https://www.zotero.org/google-docs/?w0wh7m) is a visuo-spatial working memory task based on the Corsi Block Span Test [(Corsi, 1972)](https://www.zotero.org/google-docs/?Rc52xB).. Eight randomly arranged squares are displayed on the screen, and each square flashes to a different color in a predetermined sequence. Participants are asked to repeat the sequence, which progresses from 2 to 8 squares, and then are asked to repeat another sequence in reverse. The outcome measures used in the present study were the highest sequence and total correct for both the forward and backward trials, as have previously been analyzed in this sample [(Meyers et al., 2019)](https://www.zotero.org/google-docs/?TWtMxI). These measures were not transformed.

The 3,681 participants included in the present analyses completed 4.20±2.08 neuropsychological assessment waves (range = 0-8), with minor differences across measures.

**Analyses**

All stage-based phenotypes for EXT and INT were entered into models simultaneously, as were all alcohol milestones in predicting stage-based phenotypes; given sample size differences in EF measures, these were analyzed separately from the EXT and INT models. All PGS were included together in all PGS models.

**Justification for use of dichotomous phenotypes:** Although reliance on thresholds of diagnosis/no diagnosis may obfuscate important heterogeneity and varying severity within diagnostic categories, such dichotomous categorizations were used for two reasons. The primary reason is that many participants entered the COGA Prospective Study after the onsets of alcohol involvement and other diagnoses, so using retrospectively reported ages of onset was the only way to examine longitudinal relationships while minimizing substantial loss of data. Although the COGA Prospective Study collects continuous measures of internalizing and externalizing behavior (e.g., the Adult Self Report), most individuals have fewer than 2 waves of such data, considerably limiting the extent to which prospective relationships could be examined. The second reason is that the survival models take into account that some participants may not yet meet diagnostic criteria for some outcome but may at some point in the future, allowing us to use data from all individuals; these models necessitate dichotomous outcomes.

***Covarying for age of first drink and first intoxication.*** In models analyzing survival from first drink to other alcohol outcomes, the age of first drink was included as a covariate in the form of a categorical variable of ages of initiation: ≤ 12, 13, 14, 15, 16, 17, 18, ≥ 19. Age groups were combined when their associations are statistically indistinct (i.e., overlapping confidence intervals), and the median age (i.e., 16) was the reference group. Similarly, in models exploring survival from first intoxication to first AUD symptom and first AUD diagnosis, age of first intoxication was included as a covariate: ≤ 14, 15, 16, 17, 18, 19, 20, ≥ 21; age 17 was the median and formed the reference group. These covariates were included to adjust for any variability in risk period, following (Bucholz et al., 2017).

Models with EF phenotypes as predictors and alcohol transitions as outcomes additionally covaried for age at EF assessment and the time from EF assessment to  difference between that age and the age of the milestone age (or of exit from the study).

***Time-varying data.*** In models exploring survival to alcohol milestones, stage-based behavioral phenotypes were coded as time-varying, with 0s present in each row until onset of the particular symptom or diagnosis, and 1s thereafter. For participants reporting the same ages of onset for alcohol milestones (e.g., age 16 for both initiation and intoxication), “0” times were recoded to a small number less than 1 to allow models to run without unnecessary loss of data. Due to variability in retrospective reporting of symptom onsets, the first-reported onsets were used given closer proximity to the true time point.

***Proportional hazards assumption.*** Violations of the proportional hazards assumption (i.e., that the hazard associated with a predictor remains proportional over time) were investigated using the cox.zph() function, which assesses Shoenfeld residuals and indicates which predictors are responsible for violations. Violations were resolved by incorporating age interactions with the particular variable in the model, which were chosen based on divergence of hazards on graphs of the residuals or of the “failure rates.”

***Severity analyses.*** Given heterogeneity in the severity of AUD based on the number of symptoms endorsed, follow-up analyses examined associations between stage-based phenotypes and hazards of transition from first drink to first mild, moderate, and severe AUD diagnosis. Individuals may have met criteria for AUD at different severity levels across assessments, and such individuals were included in multiple analyses. As with primary analysis, FDR correction was applied across all internalizing phenotypes across the 3 outcome variables, all externalizing phenotypes, all internalizing PGS, all externalizing PGS, and all EF PGS, separately.

***Executive function trajectories.*** Linear mixed effects models were used to test whether alcohol milestone interacted with age to predict each of seven metrics of EF performance. That is, we tested whether milestone moderated the slope of EF performance over time. Specifically, milestone was a 5-level categorical variable denoting the “most severe” stage of alcohol involvement met by a particular wave, with “0” = no milestone yet, “1” = individual has initiated drinking, “2” = individual has become intoxicated on alcohol, “3” = individual has endorsed a symptom of AUD, “4” = individual has met diagnostic criteria for AUD. Age was separated into within- (i.e., person-mean-centered) and between-person (i.e., person-means of age) effects to account for both change within person as well as the relatively wide spread of ages across individuals. Linear and quadratic effects of both age variables were included.

The analysis proceeded in several steps per EF variable:

1. To test for main effects of milestone, a model in which main effects of age and milestone were included alongside covariates and random intercepts was compared to a model excluding the effect of milestone, e.g.:

Mod1 <- lmer(log2(ToL_apt) ~ (age.pm + age.pm^2^)*(age.pmc + age.pmc^2^ ) + covariates + (1 + age.pmc | ID) + (1 | famID), data = data)

Mod2 <- lmer(log2(ToL_apt) ~ milestone + (age.pm + age.pm^2^)*(age.pmc + age.pmc^2^ ) + covariates + (1 + age.pmc | ID) + (1 | famID), data = data)

anova(Mod1, Mod2)

**age.pm** = person-mean age, grand-mean-centered

**age.pmc** = person-mean-centered age

**ToL_apt** = Tower of London average pickup time

2. To test for the overall effect of interactions (i.e., the omnibus test), a model in which milestone was interacted with all 4 age variables was compared to a model in which only main effects were included, e.g.:

Mod3 <- lmer(log2(ToL_apt) ~ milestone*(age.pm + age.pm^2^ + age.pmc + age.pmc^2^ ) + (age.pm + age.pm^2^)*(age.pmc + age.pmc^2^ ) + covariates + (1 + age.pmc | ID) + (1 | famID), data = data)

anova(Mod2, Mod3)

3. If the model comparisons in Steps 2-3 were significant following FDR correction for 14 tests, post-hoc pairwise comparison tests were conducted following identification and examination of influential data points at level 2 (i.e., the individual) using cook’s distance. Influential data points were examined for potential oddities in the data and were removed from analyses if any were identified and/or BIC model comparisons revealed meaningful differences. Contrasts were specified and tested using the glht() function from the multcomp package in R [(Hothorn et al., 2023)](https://www.zotero.org/google-docs/?KiNGtU) to test whether milestone groups differed in means (if omnibus tests in Step 2 were significant) and/or quadratic slopes (if omnibus tests in Step 3 were significant). All milestone groups were compared to each other (n=10 comparisons), and in additionally, “0” was compared to all other milestones, “1” was compared to milestones 2 through 4, “0” and “1” were compared to milestones 2-4, and milestones 0-2 were compared to milestones 3-4 in order to further assess differences between earlier and later stages.

Covariates included sex, race, ethnicity, highest parent education, parent household income, case/control family status, parent AUD, parent separation between ages 12-17, birth cohort, study site, and tobacco and marijuana initiation and dependence.

The same procedure was repeated for PGS analyses. Specifically, a model with covariates, age effects, and alcohol PGS (drinks per week and problematic alcohol use) was compared to a model excluding alcohol PGS, and a model including interactions between alcohol PGS and age variables was compared to a main-effects only model. FDR correction was conducted across the resulting 14 p-values. Covariates included sex, highest parent education, parent household income, case/control family status, parent AUD, parent separation between ages 12-17, birth cohort, study site, the first 10 ancestrally informative principal components, and non-alcohol PGS (i.e., MDD, GAD, Neuroticism, ADHD, Risk Tolerance, and common EF).

***Power Analysis.*** In order to determine whether sufficient data were present to examine associations between alcohol milestones and future stage-based symptom onsets, power analyses were conducted using the powerSurvEpi package’s powerCT() function in R [(Qiu et al., 2021)](https://www.zotero.org/google-docs/?5eD9Ag). A person-period data set was generated for each outcome and included in the powerCT() function along with a survival function, the number of participants in the “experimental group” (i.e., those who have initiated alcohol use, become intoxicated,  have an AUD symptom, or have an AUD diagnosis) and in the “control group” (i.e., those that have not experienced these alcohol milestones), and an alpha level (.05). Different hazard ratio estimates were input in the function until 80% power was reached.

***Examination of Multicollinearity.*** Given the number of predictors included in analyses, correlations among predictors and indices of multicollinearity were computed. The highest correlations among INT and EXT phenotypes (examined using Cramer’s V) were between ADHD hyperactive and inattentive symptoms (0.57), major depressive episode and suicidal ideation (0.44), and conduct disorder and oppositional defiant disorder symptoms (0.41). The highest correlations (r) among PGS were between Major Depressive Disorder and Neuroticism PGS (0.48) and between Drinks per Week and Problematic Alcohol Use PGS (0.36). Variance Inflation Factor (VIF) values—which measure the degree of inflation of variance of parameter estimates due to multicollinearity (Kutner et al., 2005)—for all primary predictors for all models were well below the rule-of-thumb threshold of 5 (Kim, 2019), suggesting that there is no problematic multicollinearity present in our models. Specifically, VIF values ranged from 1.054 to 1.654 for phenotypic models of alcohol transitions, from 1.088 to 1.689 for GS models of alcohol transitions, from 1.126 to 2.881 for phenotypic models of stage-based symptom onsets, and from 1.076 to 1.588 for PGS models of stage-based symptom onsets.

The highest correlations (Cramer’s V) among EXT and INT predictors were between ADHD hyperactive and inattentive symptoms (0.57), major depressive episode and suicidal ideation (0.44), and conduct disorder and oppositional defiant disorder symptoms (0.41). The highest correlations (r) among PGS were between Major Depressive Disorder and Neuroticism PGS (0.48) and between Drinks per Week and Problematic Alcohol Use PGS (0.36). VIF values for all predictors for all models were well below the rule-of-thumb threshold of 5 (Kim, 2019), suggesting that there is no problematic multicollinearity present in our models.

**Genotyping, Quality Control, and Polygenic Score (PGS) Computation.**

***Discovery GWAS.*** Discovery GWAS (with the COGA sample left out of GWAS-generated summary statistics, when relevant) used to compute PGS in the European ancestry subsample included **Alcohol phenotypes (i.e., *Drinks/Week*** [DPW; EA: n=666,978; AFR n=8,078, supplemented with n=666,978 EA; [Saunders et al., 2022](https://www.zotero.org/google-docs/?518Chx)], ***Problematic Alcohol Use*** [PAU; EA n=435,563, [Zhou et al., 2020](https://www.zotero.org/google-docs/?EyjwvY); AFR n=56,648 supplemented with n=351,113 EA, [Kranzler et al., 2019](https://www.zotero.org/google-docs/?oMH47E)], **Internalizing (i.e., *Major Depressive Disorder*** [MDD; EA n=750,414, [Howard et al., 2019; Levey et al., 2021](https://www.zotero.org/google-docs/?ayQ196); AFR n=59,600 supplemented with n=570,414 EA, [Levey et al., 2021](https://www.zotero.org/google-docs/?ilYuUP)], ***Generalized Anxiety Disorder*** [GAD; EA n=175,163, [Levey et al., 2020](https://www.zotero.org/google-docs/?pYtL7A)], ***Neuroticism*** [EA n=390,278; [Nagel et al., 2018](https://www.zotero.org/google-docs/?YEbrTa)], **Externalizing (i.e., *Risk Tolerance*** [EA n=466,571, [Karlsson Linnér et al., 2019](https://www.zotero.org/google-docs/?mUO8YH)], ***ADHD*** [EA n=55,374, [Demontis et al., 2019](https://www.zotero.org/google-docs/?mGP8Co)], ***Common Executive Function*** [cEF; EA n=427,037, [Hatoum et al., 2023](https://www.zotero.org/google-docs/?n5UYnO)]).

These GWAS were selected based on sample size, relevance to the neurobiological stage-based model, and the present study’s reliance on psychiatric phenotypes to test hypotheses related to this model. DPW and PAU represent the best-powered, published GWAS of alcohol consumption and problematic use, respectively. Selection of MDD, ADHD, and Executive Function GWAS was based on similarity to measured phenotypes available in the COGA Prospective Study. No well-powered GWAS of Social Anxiety Disorder, Panic Disorder, Agoraphobia, Suicidal Ideation, Oppositional Defiant Disorder, or Conduct Disorder were published at the time the present analyses were completed. As such, the decision was made to utilize well-powered GWAS of other phenotypes mapping onto internalizing and externalizing traits: Generalized Anxiety Disorder, Neuroticism, and Risk Tolerance, which are not assessed or are not assessed in two or more waves in the COGA Prospective Study.

***Genotyping, Quality Control, and Imputation.*** The Illumina 1 M and Illumina OmniExpress, and Smokescreen arrays were used for genotyping. A set of 47,000 common (MAF>10%), independent (R^2^<0.5), high quality (missingness<2% and HWE p-values>0.001) variants genotyped on all arrays were selected, identity-by-descent was calculated using PLINK [(Chang et al., 2015; Purcell et al., 2007)](https://www.zotero.org/google-docs/?WqIq7G), and duplicates were removed and reported pedigree structures confirmed. SNP genotypes were tested for Mendelian inconsistencies (PedCheck; [O’Connell & Weeks, 1998)](https://www.zotero.org/google-docs/?IKZLV5) with this revised family structure. Genotype inconsistencies were set to missing. Principal components (PCs) were calculated with Eigenstrat [(Price et al., 2006)](https://www.zotero.org/google-docs/?8kM6D8) using the 47,000 variants and data from the 1000 Genomes (Phase 3, version 5; [Auton et al., 2015)](https://www.zotero.org/google-docs/?H2WJFI). Each individual was first assigned to African ancestry (AFR), European ancestry (EA), or Other based on the first two PCs and then assigned to a family-based ancestral population according to the majority of individual-based assignments in that family. A total of 8038 European ancestry samples and 3654 African ancestry samples were ultimately included.

Imputation was conducted on non-palindromic variants with missing rates <5%, MAF>3%, and HWE p-values >0.0002, separately by array, using SHAPEIT2 [(Delaneau et al., 2013)](https://www.zotero.org/google-docs/?2MHpUl) and Minimac3 [(Das et al., 2016)](https://www.zotero.org/google-docs/?FtRf7j). Samples of European ancestry were imputed to the Haplotype Reference Consortium (HRC; [McCarthy et al., 2016)](https://www.zotero.org/google-docs/?ohP0QE) reference panel using the Michigan imputation server, and samples of African ancestry were imputed using the Consortium on Asthma among African-Ancestry Populations in the Americas (CAAPA; [Mathias et al., 2016)](https://www.zotero.org/google-docs/?3yX4ZX) also using the Michigan imputation server. SNPs were then filtered to only include those with INFO scores > 0.8, minor allele frequency > 0.01 and that passed Hardy-Weinberg equilibrium (HWE p<10^−6^). See Johnson et al. (2023) for further information.

***Polygenic score (PGS) computation.*** PGS were computed in the COGA prospective sample using PGS-CS [(Ge et al., 2019)](https://www.zotero.org/google-docs/?EG9dbJ), a Bayesian regression and continuous shrinkage method, and with PGS-CSx [(Ruan et al., 2022)](https://www.zotero.org/google-docs/?JpcoQF), an extension of PGS-CS that integrates summary statistics and LD reference panels from multiple ancestral groups to enhance polygenic prediction across populations for which sufficiently powered GWAS may not exist. Effect sizes came from the summary statistics from GWAS referenced above, and Linkage Disequilibrium information came from the 1000 Genomes Phase 3 European and African reference panels. The “auto” and “meta” options were used for PGS-CS and PGS-CSx for European and African-American ancestry, respectively. The “meta” option in PRS-CSx uses an inverse-variance-weighted meta-analysis in the Gibbs sampler to integrate population-specific posterior SNP effect sizes (Ruan et al., 2022). PRS-CSx leverages summary statistics and reference panels from other ancestry groups (in this case, European ancestry) to improve prediction in populations for which GWAS are not sufficiently powered (in this case, African ancestry). As such, for the three phenotypes for which African ancestry summary statistics were available, European ancestry summary statistics of the same or similar phenotype were used to boost prediction, as noted above in the description of GWAS used.

**Supplemental Results**

**Transitions from first intoxication to first AUD symptom and first AUD diagnosis.** Conduct disorder symptoms were associated with quicker progression from first intoxication to first AUD symptom and first AUD diagnosis (HR≥1.19, *p*≤4.50e-03, *p_FDR_*≤0.012). Hyperactive ADHD symptoms were further associated with faster transition from first intoxication to first AUD diagnosis (HR=1.34, *p*=5.00e-05, *p_FDR_*=4.00e-04). No other associations survived FDR correction (HR=0.92-1.21 , *p*≥0.043, *p_FDR_*≥0.086; **Supplementary Table 4**).

PGS for generalized anxiety disorder were significantly associated with hazards of progression from first intoxication to first AUD symptom (HR=1.10, *p*=4.10E-03, *p_FDR_*=0.025). No other stage-based PGS were significantly associated with hazards of progression from first intoxication to first AUD symptom or first AUD diagnosis (HR=0.93-1.09, *p*≥0.018, *p_FDR_*≥0.054; **Supplementary Table 10**). MDD PGS was not associated with these transitions among individuals of African-American ancestry (HR=1.00, *p*≥0.94)

**Results Stratified by PCA-selected ancestry group.**

***EXT and INT Predicting Alcohol Transitions***

**European ancestry*.*** Results in the European ancestry subgroup largely mirrored those in the full sample, with conduct disorder and ADHD hyperactive symptoms showing the most consistent associations with risk for alcohol transitions and internalizing symptoms showing largely null associations. Following FDR correction for all EXT associations, ADHD hyperactive symptoms prior to age 13 were significantly associated with increased hazards for subsequent first drink (HR=1.93, *p*=5.80e-07, *p_FDR_*=1.16e-05). ADHD hyperactive symptoms at any age and conduct disorder symptoms were further associated with increased hazards for onset of AUD symptoms and AUD diagnosis following alcohol initiation (HR≥1.23, *p*≤8.10e-03, *p_FDR_*≤0.032). No other associations survived multiple testing correction (**Supplementary Table 7**).

**African-American ancestry*.*** Among individuals of PCA-selected African-American ancestry, conduct disorder symptoms were associated with increased hazards of initiating alcohol use and transitioning to first intoxication (HR≥1.32, *p*≤7.90e-03, *p_FDR_*≤0.031), and oppositional defiant symptoms prior to age 15 were associated with hazards of initiating use (HR=1.64, *p*=1.60e-03, *p_FDR_*=0.027). No other associations between stage-based phenotypes and alcohol transitions survived FDR correction (HR=0.70-1.62, *p*≥0.017, *p_FDR_*≥0.065). The association with the largest effect size and smallest p-value was between oppositional defiant disorder symptoms prior to age 15 and time to first drink (**Supplementary Table 7**).

***Alcohol Milestones Predicting Later EXT and INT***

**European ancestry*.*** Following FDR correction for all tests, alcohol initiation was associated with subsequent MDE (specific to ages 15 or younger), suicidal ideation, and conduct disorder symptoms (HR≥1.67, *p*≤2.50e-04, *p_FDR_*≤2.08e-03), but not with oppositional defiant, panic, or social anxiety disorder symptoms (HR≤1.63, *p*≥0.053). No other associations survived FDR correction (HR=0.31-2.09, *p*≥0.017, *p_FDR_*≥0.10; **Supplementary Table 8**).

**African-American ancestry*.*** No associations between alcohol milestones and onsets of stage-based symptoms survived FDR correction (HR=0.31-2.59, *p*≥8.10e-03, *p_FDR_*≥0.19). Notably, the models predicting panic, oppositional defiant, and social anxiety disorder symptom onset did not converge, so those estimates should be interpreted with caution (**Supplementary Table 9**).

**Severity analyses.**

Externalizing symptoms were associated with increased hazards for transition from first drink to all AUD diagnoses, with conduct disorder symptoms linked to first moderate and first severe AUD (HR≥1.48, *p*≤8.60e-04, *p*_FDR_≤3.30e-03), hyperactive ADHD symptoms just to first mild AUD (HR=1.40, *p*=1.70e-05, *p*_FDR_=2.04e-04), and oppositional defiant symptoms to first severe AUD (HR=1.63, *p*=1.10e-03, *p*_FDR_=3.30e-03). Internalizing (i.e., social anxiety and suicidal ideation) were associated with increased hazards for progression to severe AUD (HR≥1.55, *p*≤1.60e-03, *p*_FDR_≤0.012). Notably, the size of association between major depressive episode and hazards for severe AUD (HR=1.08, *p*=0.58) increased substantially when suicidal ideation was removed from the model (HR=1.25, *p*=0.10). No other stage-based phenotypes were related to hazards for AUD diagnoses (HR=0.93-1.51, *p*≥0.018, *p*_FDR_≤0.090; **Figure 2; Supplementary Table 2**).

Polygenic propensity to higher executive function was associated with increased hazards for the transition to first mild diagnosis (HR=1.09, *p*=0.026, *p*_FDR_=0.039) and with reduced hazards for the transition to first severe AUD (HR=0.85, *p*=0.039, *p*_FDR_=0.045). MDD PGS was nominally associated with reduced hazards for the transition to first mild AUD (HR=0.90, *p*=0.034, *p*_FDR_=0.15), and GAD PGS was nominally linked with increased hazards for the transition to first severe AUD diagnosis (HR=1.25, *p*=6.60e-03, *p*_FDR_=0.059). No other PGS were significantly linked to progression to AUD diagnosis at any level of severity (HRs=0.82-1.09, *p*s≥0.058, *ps*_FDR_≥0.17; **Figure 7; Supplementary Table 11**)

**Executive function trajectories.**

There were no significant omnibus tests for the main effect of milestone as well as the overall effect of interactions between milestone and linear and quadratic effects of person-mean-age and time-in-study (i.e., person-mean-centered age) on executive function phenotypes (*p*≥3.97e-03, *p*_FDR_≥0.058; **Supplementary Table 6**).

Alcohol PGS did not show any significant associations with EF beyond age and covariate effects, nor did they significantly interact with age variables (*p*≥0.107; **Supplementary Table 13**)

**Power Analysis. Supplementary Tables 14-15** show the effect sizes required to achieve 80% to detect associations between stage-based phenotypes and alcohol milestones. There was 80% power to detect hazard ratios between stage-based phenotypes and subsequent alcohol transitions of 1.13-1.26 for initiation, 1.15-1.41 for intoxication, 1.25-1.63 for AUD symptom(s), and 1.47-2.77 for AUD diagnosis (**Supplementary Table 14**). There was 80% power to detect hazard ratios between alcohol milestones and subsequent stage-based outcomes of 1.16-1.19 for MDE, 1.20-1.26 for suicidal ideation, 1.36-1.65 for panic disorder, 1.46-1.70 for social anxiety disorder, 1.54-1.94 for agoraphobia, 1.23-1.32 for conduct disorder symptoms, and 1.24-1.29 for oppositional defiant disorder symptoms (**Supplementary Table 15**).

**Supplementary Table 1.** Cox Proportional Hazard Model Results for Stage-Based Phenotypes and Alcohol Transitions.

| **Phenotype** | **HR [95% CI]** | **p** | **p_FDR_** |
| --- | --- | --- | --- |
| **Time to First Drink** | | | |
| Major Depressive Episode | 1.00 [0.89, 1.13] | 0.95 | 0.95 |
| Social Anxiety Disorder Sxs | 0.97 [0.76, 1.23] | 0.80 | 0.95 |
| Panic Disorder Sxs | 0.84 [0.66, 1.07] | 0.17 | 0.53 |
| Agoraphobia Sxs | 0.82 [0.60, 1.11] | 0.20 | 0.53 |
| Suicidal Ideation | 1.03 [0.90, 1.19] | 0.64 | 0.91 |
| Inattentive ADHD Sxs | 1.05 [0.93, 1.18] | 0.43 | 0.54 |
| Hyperactive ADHD Sxs (age ≤11) | 1.92 [1.48, 2.49] | 8.20E-07 | 1.56E-05 |
| Hyperactive ADHD Sxs (age ≥12) | 1.16 [1.03, 1.31] | 0.012 | 0.025 |
| Oppositional Defiant Disorder Sxs | 1.24 [1.07, 1.44] | 3.50E-03 | 9.03E-03 |
| Conduct Disorder Sxs | 1.25 [1.10, 1.42] | 7.00E-04 | 2.66E-03 |
| **First Drink to First Intoxication** | | | |
| Major Depressive Episode | 1.05 [0.94, 1.16] | 0.38 | 0.76 |
| Social Anxiety Disorder Sxs | 1.14 [0.93, 1.40] | 0.21 | 0.53 |
| Panic Disorder Sxs | 0.99 [0.81, 1.21] | 0.90 | 0.95 |
| Agoraphobia Sxs | 0.91 [0.70, 1.19] | 0.50 | 0.77 |
| Suicidal Ideation | 1.04 [0.93, 1.17] | 0.46 | 0.77 |
| Inattentive ADHD Sxs | 0.93 [0.84, 1.03] | 0.17 | 0.26 |
| Hyperactive ADHD Sxs (≤1 year) | 1.02 [0.91, 1.13] | 0.77 | 0.77 |
| Hyperactive ADHD Sxs (≥2 years) | 1.15 [0.93, 1.41] | 0.19 | 0.26 |
| Oppositional Defiant Disorder Sxs | 0.96 [0.85, 1.09] | 0.54 | 0.60 |
| Conduct Disorder Sxs | 1.19 [1.07, 1.33] | 1.30E-03 | 4.12E-03 |
| **First Drink to First AUD Symptom** | | | |
| Major Depressive Episode | 0.93 [0.82, 1.04] | 0.20 | 0.53 |
| Social Anxiety Disorder Sxs | 1.18 [0.94, 1.48] | 0.16 | 0.53 |
| Panic Disorder Sxs | 0.99 [0.79, 1.23] | 0.90 | 0.95 |
| Agoraphobia Sxs | 0.99 [0.74, 1.32] | 0.95 | 0.95 |
| Suicidal Ideation | 1.11 [0.98, 1.26] | 0.11 | 0.53 |
| Inattentive ADHD Sxs | 0.92 [0.81, 1.04] | 0.18 | 0.26 |
| Hyperactive ADHD Sxs | 1.16 [1.03, 1.31] | 0.014 | 0.027 |
| Oppositional Defiant Disorder Sxs | 1.05 [0.91, 1.22] | 0.49 | 0.58 |
| Conduct Disorder Sxs | 1.28 [1.13, 1.44] | 6.10E-05 | 2.90E-04 |
| **First Drink to First AUD Diagnosis** | | | |
| Major Depressive Episode | 0.95 [0.82, 1.09] | 0.44 | 0.77 |
| Social Anxiety Disorder Sxs | 1.13 [0.87, 1.47] | 0.35 | 0.76 |
| Panic Disorder Sxs | 1.18 [0.93, 1.49] | 0.17 | 0.53 |
| Agoraphobia Sxs | 1.01 [0.73, 1.40] | 0.93 | 0.95 |
| Suicidal Ideation | 1.16 [1.00, 1.35] | 0.043 | 0.53 |
| Inattentive ADHD Sxs | 0.89 [0.77, 1.03] | 0.13 | 0.23 |
| Hyperactive ADHD Sxs | 1.37 [1.19, 1.58] | 1.80E-05 | 1.14E-04 |
| Oppositional Defiant Disorder Sxs (≤1 year) | 1.37 [1.11, 1.69] | 3.80E-03 | 9.03E-03 |
| Oppositional Defiant Disorder Sxs (≥2 years) | 0.97 [0.79, 1.19] | 0.76 | 0.77 |
| Conduct Disorder Sxs | 1.39 [1.21, 1.60] | 3.90E-06 | 3.71E-05 |

*Note.* Sxs = Symptoms. p_FDR_ reflects false discovery rate-corrected p-values across all 20 tests for internalizing (i.e., major depressive episode, social anxiety disorder symptoms, panic disorder symptoms, agoraphobia symptoms, and suicidal ideation) and, separately, across all 19 tests for externalizing (i.e., inattentive and hyperactive ADHD symptoms, oppositional defiant disorder symptoms, and conduct disorder symptoms).

**Supplementary Table 2.**  Post-hoc Results: Time from First Drink to First Mild, Moderate, and Severe AUD

| **Phenotype** | **HR [95% CI]** | **p** | **p_FDR_** |
| --- | --- | --- | --- |
| **Time from First Drink to First Mild AUD Diagnosis** | | | |
| Major Depressive Episode | 0.89 [0.76, 1.03] | 0.11 | 0.39 |
| Social Anxiety Disorder Sxs | 0.91 [0.67, 1.22] | 0.52 | 0.78 |
| Panic Disorder Sxs | 0.9 [0.69, 1.16] | 0.40 | 0.78 |
| Agoraphobia Sxs | 0.88 [0.61, 1.26] | 0.47 | 0.78 |
| Suicidal Ideation | 1.13 [0.96, 1.32] | 0.13 | 0.39 |
| Inattentive ADHD Sxs | 0.88 [0.75, 1.02] | 0.097 | 0.17 |
| Hyperactive ADHD Sxs | 1.40 [1.2, 1.63] | 1.70E-05 | 2.04E-04 |
| Oppositional Defiant Disorder Sxs | 0.88 [0.74, 1.06] | 0.18 | 0.27 |
| Conduct Disorder Sxs | 1.19 [1.02, 1.39] | 0.025 | 0.060 |
| **Time from First Drink to First Moderate AUD Diagnosis** | | | |
| Major Depressive Episode | 0.99 [0.8, 1.24] | 0.95 | 0.98 |
| Social Anxiety Disorder Sxs | 0.93 [0.62, 1.4] | 0.73 | 0.91 |
| Panic Disorder Sxs | 1.52 [1.07, 2.15] | 0.018 | 0.090 |
| Agoraphobia Sxs | 1.21 [0.75, 1.95] | 0.44 | 0.78 |
| Suicidal Ideation | 1.11 [0.88, 1.4] | 0.38 | 0.78 |
| Inattentive ADHD Sxs | 0.99 [0.78, 1.25] | 0.91 | 0.91 |
| Hyperactive ADHD Sxs | 1.23 [0.98, 1.55] | 0.073 | 0.15 |
| Oppositional Defiant Disorder Sxs | 1.18 [0.92, 1.52] | 0.2 | 0.27 |
| Conduct Disorder Sxs | 1.48 [1.19, 1.84] | 4.80E-04 | 2.88E-03 |
| **Time from First Drink to First Severe AUD Diagnosis** | | | |
| Major Depressive Episode | 1.08 [0.82, 1.43] | 0.58 | 0.79 |
| Social Anxiety Disorder Sxs | 2.25 [1.50, 3.36] | 8.50E-05 | 1.28E-03 |
| Panic Disorder Sxs | 1.06 [0.68, 1.65] | 0.8 | 0.92 |
| Agoraphobia Sxs | 1.01 [0.57, 1.77] | 0.98 | 0.98 |
| Suicidal Ideation | 1.55 [1.18, 2.04] | 1.60E-03 | 0.012 |
| Inattentive ADHD Sxs | 1.16 [0.87, 1.56] | 0.31 | 0.37 |
| Hyperactive ADHD Sxs | 1.10 [0.82, 1.47] | 0.52 | 0.57 |
| Oppositional Defiant Disorder Sxs | 1.63 [1.22, 2.19] | 1.10E-03 | 3.30E-03 |
| Conduct Disorder Sxs | 1.60 [1.21, 2.11] | 8.60E-04 | 3.30E-03 |

*Note.* Sxs = Symptoms. p_FDR_ reflects false discovery rate-corrected p-values across all 15 internalizing and, separately, all 12 externalizing tests.

**Supplementary Table 3.** Cox Proportional Hazard Model Results for Executive Function Phenotypes and Alcohol Transitions

| **Phenotype** | **HR [95% CI]** | **p** | **p_FDR_** |
| --- | --- | --- | --- |
| **Time to First Drink** | | | |
| ToL Excess Moves ^a^ | 1.04 [0.99, 1.10] | 0.095 | 0.38 |
| ToL Average Pickup Time ^b^ | 1.02 [0.87, 1.21] | 0.79 | 0.88 |
| ToL Average Total Time ^b^ | 1.07 [0.89, 1.28] | 0.47 | 0.62 |
| VST Forward Span | 0.97 [0.92, 1.02] | 0.20 | 0.49 |
| VST Backward Span | 1.02 [0.96, 1.09] | 0.45 | 0.62 |
| VST Forward Correct | 0.98 [0.96, 1.01] | 0.21 | 0.49 |
| VST Backward Correct | 1.02 [0.98, 1.05] | 0.38 | 0.62 |
| **Time to First Intoxication** | | | |
| ToL Excess Moves ^a^ | 1.02 [0.98, 1.07] | 0.38 | 0.62 |
| ToL Average Pickup Time ^b^ | 1.13 [0.97, 1.31] | 0.13 | 0.44 |
| ToL Average Total Time ^b^ | 1.19 [1.00, 1.40] | 0.046 | 0.32 |
| VST Forward Span | 0.99 [0.94, 1.03] | 0.60 | 0.73 |
| VST Backward Span | 1.00 [0.95, 1.06] | 0.86 | 0.93 |
| VST Forward Correct | 0.99 [0.97, 1.02] | 0.49 | 0.62 |
| VST Backward Correct | 1.00 [0.97, 1.03] | 0.95 | 0.97 |
| **Time to First AUD Symptom** | | | |
| ToL Excess Moves ^a^ | 1.05 [1.00, 1.10] | 0.058 | 0.32 |
| ToL Average Pickup Time ^b^ | 1.15 [0.98, 1.35] | 0.080 | 0.37 |
| ToL Average Total Time ^b^ | 1.19 [1.00, 1.42] | 0.055 | 0.32 |
| VST Forward Span | 0.98 [0.93, 1.02] | 0.32 | 0.62 |
| VST Backward Span | 1.01 [0.95, 1.07] | 0.79 | 0.88 |
| VST Forward Correct | 0.98 [0.96, 1.01] | 0.25 | 0.54 |
| VST Backward Correct | 1.01 [0.98, 1.05] | 0.44 | 0.62 |
| **Time to First AUD Diagnosis** | | | |
| ToL Excess Moves ^a^ | 1.02 [0.97, 1.08] | 0.48 | 0.62 |
| ToL Average Pickup Time ^b^ | 1.24 [1.03, 1.50] | 0.026 | 0.32 |
| ToL Average Total Time ^b^ | 1.28 [1.03, 1.59] | 0.023 | 0.32 |
| VST Forward Span | 0.97 [0.92, 1.03] | 0.34 | 0.62 |
| VST Backward Span | 1.05 [0.98, 1.13] | 0.16 | 0.45 |
| VST Forward Correct | 1.00 [0.97, 1.03] | 0.97 | 0.97 |
| VST Backward Correct | 1.03 [0.99, 1.07] | 0.14 | 0.44 |

*Note.* ToL = Tower of London. VST = Visual Span Test. p_FDR_ reflects false discovery rate-corrected p-values across all 28 tests. ^a^ ToL Excess Moves was log2 transformed after adding “1” to the value. ^b^ These measures were log2 transformed.

**Supplementary Table 4.** Cox Proportional Hazard Model Results for Stage-Based Phenotypes and Transitions from First Intoxication

| **Phenotype** | **HR [95% CI]** | **p** | **p_FDR_** |
| --- | --- | --- | --- |
| **First Intoxication to First AUD Symptom** | | | |
| Major Depressive Episode | 0.94 [0.84, 1.06] | 0.31 | 0.62 |
| Social Anxiety Disorder Sxs | 1.21 [0.97, 1.51] | 0.088 | 0.29 |
| Panic Disorder Sxs | 1.02 [0.83, 1.26] | 0.85 | 0.85 |
| Agoraphobia Sxs | 1.06 [0.80, 1.40] | 0.68 | 0.76 |
| Suicidal Ideation | 1.13 [1.00, 1.29] | 0.049 | 0.26 |
| Inattentive ADHD Sxs | 0.96 [0.85, 1.08] | 0.50 | 0.57 |
| Hyperactive ADHD Sxs | 1.13 [1.00, 1.27] | 0.043 | 0.086 |
| Oppositional Defiant Disorder Sxs | 1.03 [0.89, 1.19] | 0.69 | 0.69 |
| Conduct Disorder Sxs | 1.19 [1.06, 1.34] | 4.50E-03 | 0.012 |
| **First Intoxication to First AUD Diagnosis** | | | |
| Major Depressive Episode | 0.96 [0.84, 1.11] | 0.59 | 0.76 |
| Social Anxiety Disorder Sxs | 1.07 [0.83, 1.37] | 0.61 | 0.76 |
| Panic Disorder Sxs | 1.16 [0.92, 1.46] | 0.21 | 0.53 |
| Agoraphobia Sxs | 1.14 [0.83, 1.56] | 0.42 | 0.70 |
| Suicidal Ideation | 1.15 [1.00, 1.33] | 0.052 | 0.26 |
| Inattentive ADHD Sxs | 0.92 [0.80, 1.06] | 0.25 | 0.33 |
| Hyperactive ADHD Sxs | 1.34 [1.16, 1.54] | 5.00E-05 | 4.00E-04 |
| Oppositional Defiant Disorder Sxs | 1.17 [0.99, 1.37] | 0.062 | 0.099 |
| Conduct Disorder Sxs | 1.32 [1.14, 1.51] | 1.20E-04 | 4.80E-04 |

*Note.* Sxs = Symptoms. p_FDR_ reflects false discovery rate-corrected p-values across all 10 tests for internalizing (i.e., major depressive episode, social anxiety disorder symptoms, panic disorder symptoms, agoraphobia symptoms, and suicidal ideation) and, separately, across all 8 tests for externalizing (i.e., inattentive and hyperactive ADHD symptoms, oppositional defiant disorder symptoms, and conduct disorder symptoms).

**Supplementary Table 5.** Cox Proportional Hazard Model Results for Alcohol Milestones and Stage-Based Symptom Onsets

| **Alcohol Milestone** | **HR [95% CI]** | **p** | **p_FDR_** |
| --- | --- | --- | --- |
| **Time to First Major Depressive Episode** | | | |
| Initiation | 1.44 [1.21, 1.72] | 5.50E-05 | 9.12E-04 |
| Intoxication | 0.95 [0.78, 1.17] | 0.64 | 0.77 |
| AUD Symptom | 0.98 [0.78, 1.21] | 0.82 | 0.88 |
| AUD Diagnosis | 1.38 [1.10, 1.73] | 5.1E-03 | 0.024 |
| **Time to First Suicidal Ideation** | | | |
| Initiation | 1.52 [1.22, 1.91] | 2.10E-04 | 1.68E-03 |
| Intoxication | 1.14 [0.88, 1.48] | 0.31 | 0.48 |
| AUD Symptom | 0.79 [0.59, 1.07] | 0.13 | 0.31 |
| AUD Diagnosis | 1.57 [1.15, 2.15] | 5.00E-03 | 0.024 |
| **Time to First Panic Disorder Symptoms** | | | |
| Initiation | 1.02 [0.66, 1.58] | 0.94 | 0.94 |
| Intoxication | 1.46 [0.92, 2.32] | 0.11 | 0.29 |
| AUD Symptom | 1.27 [0.85, 1.88] | 0.24 | 0.44 |
| AUD Diagnosis | 1.27 [0.88, 1.84] | 0.20 | 0.40 |
| **Time to First Social Anxiety Disorder Symptoms** | | | |
| Initiation | 1.07 [0.57, 2.00] | 0.84 | 0.88 |
| Intoxication | 0.42 [0.18, 0.99] | 0.048 | 0.14 |
| AUD Symptom | 1.54 [0.59, 4.02] | 0.38 | 0.54 |
| AUD Diagnosis | 1.38 [0.54, 3.55] | 0.50 | 0.67 |
| **Time to First Conduct Disorder Symptoms** | | | |
| Initiation | 1.60 [1.27, 2.03] | 7.60E-05 | 9.12E-04 |
| Intoxication | 1.41 [1.03, 1.93] | 0.031 | 0.12 |
| AUD Symptom | 1.26 [0.82, 1.93] | 0.29 | 0.48 |
| AUD Diagnosis | 0.81 [0.39, 1.68] | 0.58 | 0.73 |
| **Time to First Oppositional Defiant Disorder Symptoms** | | | |
| Initiation | 1.45 [1, 2.08] | 0.047 | 0.14 |
| Intoxication | 0.77 [0.46, 1.29] | 0.32 | 0.48 |
| AUD Symptom | 1.54 [0.81, 2.93] | 0.18 | 0.39 |
| AUD Diagnosis | 1.1 [0.5, 2.43] | 0.81 | 0.88 |

*Note.* p_FDR_ reflects false discovery rate-corrected p-values across all 24 tests.

**Supplementary Table 6.** Omnibus tests of effects of alcohol milestone and alcohol milestone*age interactions in EF trajectories

| **EF phenotype** | **Model** | **npar** | **BIC** | **Chisq** | **Df** | **P** | **P_FDR_** |
| --- | --- | --- | --- | --- | --- | --- | --- |
| ToL Excess Moves | Cov + age | 39 | 36468 |  |  |  |  |
|  | Cov + age + milestone | 43 | 36494 | 11.19 | 4 | 0.0246 | 0.0588 |
|  | Cov + age*milestone | 59 | 36629 | 13.01 | 16 | 0.672 | 0.672 |
| ToL Average Pickup Time | Cov + age | 39 | 9382.9 |  |  |  |  |
|  | Cov + age + milestone | 43 | 9416.7 | 3.23 | 4 | 0.520 | 0. 672 |
|  | Cov + age*milestone | 59 | 9533.8 | 31.03 | 16 | 0.0134 | 0. 0588 |
| ToL Average Total Time | Cov + age | 39 | 6954.8 |  |  |  |  |
|  | Cov + age + milestone | 43 | 6988.4 | 3.35 | 4 | 0.501 | 0. 672 |
|  | Cov + age*milestone | 59 | 7101.5 | 35.01 | 16 | 3.97E-03 | 0. 0588 |
| VST Forward Span | Cov + age | 39 | 34056 |  |  |  |  |
|  | Cov + age + milestone | 43 | 34091 | 2.43 | 4 | 0.657 | 0. 672 |
|  | Cov + age*milestone | 59 | 34209 | 29.81 | 16 | 0.0190 | 0. 0588 |
| VST Forward Total Correct | Cov + age | 39 | 45382 |  |  |  |  |
|  | Cov + age + milestone | 43 | 45415 | 3.70 | 4 | 0.448 | 0. 672 |
|  | Cov + age*milestone | 59 | 45533 | 30.59 | 16 | 0.0152 | 0. 0588 |
| VST Backward Span | Cov + age | 39 | 28780 |  |  |  |  |
|  | Cov + age + milestone | 43 | 28814 | 2.45 | 4 | 0.651 | 0. 672 |
|  | Cov + age*milestone | 59 | 28933 | 28.82 | 16 | 0.0252 | 0. 0588 |
| VST Backward Total Correct | Cov + age | 39 | 39929 |  |  |  |  |
|  | Cov + age + milestone | 43 | 39963 | 3.17 | 4 | 0.529 | 0. 672 |
|  | Cov + age*milestone | 59 | 40087 | 23.33 | 16 | 0.105 | 0.210 |

*Note.* ToL = Tower of London Test. VST = Visual Span Test. Cov = covariates. Milestone is a 5-level categorical variable representing the “most severe” stage of alcohol involvement endorsed at a particular time point (0 = no milestone, 1 = initiation, 2 = intoxication, 3 = AUD symptom, 4 = AUD diagnosis). Npar = number of parameters in the model. Df = degrees of freedom. P-values are for model comparisons between the model and the previous model, conducted via the anova() function in R. p_FDR_ reflects FDR-corrected p-values across all 14 tests.

**Supplementary Table 7.** Cox Proportional Hazard Model Results for Stage-Based Phenotypes and Alcohol Transitions, stratified by ancestry.

| **European Ancestry** | | | |
| --- | --- | --- | --- |
| **Phenotype** | **HR [95% CI]** | **p** | **p_FDR_** |
| **Time to First Drink** | | | |
| Major Depressive Episode | 1.04 [0.88, 1.23] | 0.62 | 0.89 |
| Social Anxiety Disorder Sxs | 0.91 [0.68, 1.21] | 0.52 | 0.89 |
| Panic Disorder Sxs | 1.00 [0.73, 1.37] | 0.98 | 0.98 |
| Agoraphobia Sxs | 0.92 [0.59, 1.44] | 0.71 | 0.89 |
| Suicidal Ideation | 0.86 [0.71, 1.04] | 0.13 | 0.89 |
| Inattentive ADHD Sxs | 1.06 [0.91, 1.24] | 0.47 | 0.53 |
| Hyperactive ADHD Sxs (age ≤12) ^a^ | 1.93 [1.49, 2.50] | 5.80E-07 | 1.16E-05 |
| Hyperactive ADHD Sxs (age ≥13) | 1.18 [1.00, 1.39] | 0.050 | 0.11 |
| Oppositional Defiant Disorder Sxs | 1.15 [0.93, 1.41] | 0.20 | 0.31 |
| Conduct Disorder Sxs (age ≤12) | 1.57 [1.08, 2.27] | 0.017 | 0.057 |
| Conduct Disorder Sxs (age 13-15) | 1.19 [0.95, 1.49] | 0.14 | 0.23 |
| Conduct Disorder Sxs (age ≥16) | 0.87 [0.63, 1.19] | 0.37 | 0.53 |
| **First Drink to First Intoxication** | | | |
| Major Depressive Episode | 0.96 [0.84, 1.10] | 0.55 | 0.89 |
| Social Anxiety Disorder Sxs | 1.07 [0.84, 1.37] | 0.59 | 0.89 |
| Panic Disorder Sxs | 1.06 [0.83, 1.36] | 0.63 | 0.89 |
| Agoraphobia Sxs | 0.94 [0.67, 1.32] | 0.71 | 0.89 |
| Suicidal Ideation | 0.99 [0.85, 1.15] | 0.86 | 0.98 |
| Inattentive ADHD Sxs | 0.88 [0.77, 1.01] | 0.062 | 0.11 |
| Hyperactive ADHD Sxs | 1.02 [0.90, 1.17] | 0.73 | 0.73 |
| Oppositional Defiant Disorder Sxs | 0.97 [0.81, 1.15] | 0.72 | 0.73 |
| Conduct Disorder Sxs | 1.17 [1.01, 1.35] | 0.042 | 0.11 |
| **First Drink to First AUD Symptom** | | | |
| Major Depressive Episode | 0.93 [0.8, 1.09] | 0.36 | 0.89 |
| Social Anxiety Disorder Sxs | 1.13 [0.86, 1.49] | 0.38 | 0.89 |
| Panic Disorder Sxs | 1.08 [0.83, 1.41] | 0.56 | 0.89 |
| Agoraphobia Sxs | 0.99 [0.68, 1.42] | 0.94 | 0.98 |
| Suicidal Ideation | 0.93 [0.78, 1.09] | 0.37 | 0.89 |
| Inattentive ADHD Sxs | 0.85 [0.73, 1.00] | 0.048 | 0.11 |
| Hyperactive ADHD Sxs | 1.23 [1.06, 1.43] | 8.10E-03 | 0.032 |
| Oppositional Defiant Disorder Sxs (≤1) ^b^ | 1.24 [1;00, 1.55] | 0.055 | 0.11 |
| Oppositional Defiant Disorder Sxs (≥2) | 0.90 [0.68, 1.19] | 0.46 | 0.53 |
| Conduct Disorder Sxs | 1.26 [1.08, 1.48] | 4.00E-03 | 0.020 |
| **First Drink to First AUD Diagnosis** | | | |
| Major Depressive Episode | 0.91 [0.77, 1.09] | 0.31 | 0.89 |
| Social Anxiety Disorder Sxs | 1.17 [0.86, 1.60] | 0.32 | 0.89 |
| Panic Disorder Sxs | 1.12 [0.85, 1.49] | 0.43 | 0.89 |
| Agoraphobia Sxs | 1.03 [0.69, 1.54] | 0.89 | 0.98 |
| Suicidal Ideation | 1.05 [0.87, 1.26] | 0.62 | 0.89 |
| Inattentive ADHD Sxs | 0.93 [0.77, 1.11] | 0.41 | 0.53 |
| Hyperactive ADHD Sxs | 1.40 [1.18, 1.66] | 1.60E-04 | 1.53E-03 |
| Oppositional Defiant Disorder Sxs | 1.08 [0.87, 1.34] | 0.48 | 0.53 |
| Conduct Disorder Sxs | 1.39 [1.17, 1.66] | 2.30E-04 | 1.53E-03 |
| **African-American ancestry** | | | |
| **Phenotype** | **HR [95% CI]** | **p** | **p_FDR_** |
| **Time to First Drink** | | | |
| Major Depressive Episode | 0.97 [0.78, 1.20] | 0.77 | 0.86 |
| Social Anxiety Disorder Sxs | 1.62 [1.03, 2.55] | 0.039 | 0.51 |
| Panic Disorder Sxs | 0.70 [0.42, 1.17] | 0.17 | 0.68 |
| Agoraphobia Sxs | 0.85 [0.50, 1.43] | 0.54 | 0.80 |
| Suicidal Ideation | 1.19 [0.93, 1.54] | 0.17 | 0.68 |
| Inattentive ADHD Sxs | 1.02 [0.83, 1.25] | 0.84 | 0.95 |
| Hyperactive ADHD Sxs | 1.01 [0.83, 1.23] | 0.95 | 0.95 |
| Oppositional Defiant Disorder Sxs (age ≤14) | 1.64 [1.20, 2.22] | 1.60E-03 | 0.027 |
| Oppositional Defiant Disorder Sxs (age ≥15) | 0.92 [0.69, 1.24] | 0.60 | 0.88 |
| Conduct Disorder Sxs | 1.35 [1.10, 1.65] | 3.60E-03 | 0.031 |
| **First Drink to First Intoxication** | | | |
| Major Depressive Episode | 1.07 [0.87, 1.32] | 0.5 | 0.80 |
| Social Anxiety Disorder Sxs | 1.44 [0.93, 2.23] | 0.098 | 0.65 |
| Panic Disorder Sxs | 0.82 [0.48, 1.38] | 0.45 | 0.80 |
| Agoraphobia Sxs | 1.25 [0.72, 2.16] | 0.43 | 0.80 |
| Suicidal Ideation | 1.00 [0.79, 1.26] | 0.97 | 0.98 |
| Inattentive ADHD Sxs | 0.95 [0.76, 1.19] | 0.68 | 0.89 |
| Hyperactive ADHD Sxs | 1.03 [0.83, 1.28] | 0.8 | 0.95 |
| Oppositional Defiant Disorder Sxs | 1.10 [0.87, 1.39] | 0.43 | 0.81 |
| Conduct Disorder Sxs | 1.32 [1.08, 1.62] | 7.90E-03 | 0.045 |
| **First Drink to First AUD Symptom** | | | |
| Major Depressive Episode | 0.94 [0.73, 1.21] | 0.62 | 0.80 |
| Social Anxiety Disorder Sxs | 1.27 [0.77, 2.07] | 0.35 | 0.80 |
| Panic Disorder Sxs | 0.99 [0.54, 1.83] | 0.98 | 0.98 |
| Agoraphobia Sxs | 1.13 [0.60, 2.10] | 0.71 | 0.84 |
| Suicidal Ideation | 1.12 [0.84, 1.49] | 0.45 | 0.80 |
| Inattentive ADHD Sxs | 1.08 [0.83, 1.40] | 0.59 | 0.88 |
| Hyperactive ADHD Sxs | 1.15 [0.89, 1.48] | 0.28 | 0.68 |
| Oppositional Defiant Disorder Sxs | 1.07 [0.82, 1.41] | 0.62 | 0.88 |
| Conduct Disorder Sxs | 1.12 [0.87, 1.44] | 0.39 | 0.81 |
| **First Drink to First AUD Diagnosis** | | | |
| Major Depressive Episode | 1.12 [0.83, 1.50] | 0.46 | 0.80 |
| Social Anxiety Disorder Sxs | 1.14 [0.69, 1.89] | 0.60 | 0.80 |
| Panic Disorder Sxs | 1.19 [0.66, 2.12] | 0.57 | 0.80 |
| Agoraphobia Sxs | 1.17 [0.61, 2.24] | 0.64 | 0.80 |
| Suicidal Ideation | 1.35 [1.00, 1.82] | 0.051 | 0.51 |
| Inattentive ADHD Sxs | 0.84 [0.62, 1.14] | 0.25 | 0.68 |
| Hyperactive ADHD Sxs | 1.47 [1.07, 2.02] | 0.017 | 0.065 |
| Oppositional Defiant Disorder Sxs | 1.44 [1.06, 1.95] | 0.019 | 0.065 |
| Conduct Disorder Sxs | 0.98 [0.73, 1.32] | 0.89 | 0.95 |

*Note.* Sxs = Symptoms. p_FDR_ reflects false discovery rate-corrected p-values across all 20 internalizing and, separately, all 20 externalizing (European ancestry) tests, as well as, separately, across all 20 internalizing and, separately 17 externalizing (African-American ancestry) tests. ^a^ To address proportional hazards violations, symptoms were examined at discrete age periods. ^b^ To address proportional hazards violations, symptoms were examined at discrete numbers of years since first drink.

**Supplementary Table 8.** Cox Proportional Hazard Model Results for Alcohol Milestones and Stage-Based Symptom Onsets, stratified by ancestry

| **European Ancestry** | | | |
| --- | --- | --- | --- |
| **Alcohol Milestone** | **HR [95% CI]** | **p** | **p_FDR_** |
| **Time to First Major Depressive Episode** | | | |
| Initiation (age ≤15) ^a^ | 1.67 [1.29, 2.17] | 1.20E-04 | 1.50E-03 |
| Initiation (age ≤16) | 1.37 [0.95, 1.96] | 0.090 | 0.20 |
| Intoxication | 0.9 [0.69, 1.18] | 0.45 | 0.61 |
| AUD Symptom | 0.99 [0.75, 1.32] | 0.97 | 0.97 |
| AUD Diagnosis | 1.41 [1.06, 1.87] | 0.020 | 0.10 |
| **Time to First Suicidal Ideation** | | | |
| Initiation | 1.69 [1.28, 2.25] | 2.50E-04 | 2.08E-03 |
| Intoxication | 1.17 [0.85, 1.61] | 0.33 | 0.49 |
| AUD Symptom | 0.64 [0.44, 0.92] | 0.017 | 0.10 |
| AUD Diagnosis | 1.51 [1.03, 2.22] | 0.035 | 0.11 |
| **Time to First Panic Disorder Symptoms** | | | |
| Initiation | 1.22 [0.72, 2.06] | 0.46 | 0.61 |
| Intoxication | 1.12 [0.63, 1.97] | 0.70 | 0.75 |
| AUD Symptom | 1.69 [1.04, 2.75] | 0.036 | 0.11 |
| AUD Diagnosis | 1.42 [0.93, 2.16] | 0.10 | 0.21 |
| **Time to First Social Anxiety Disorder Symptoms** | | | |
| Initiation | 1.14 [0.56, 2.34] | 0.72 | 0.75 |
| Intoxication | 0.31 [0.11, 0.90] | 0.031 | 0.11 |
| AUD Symptom | 2.09 [0.65, 6.71] | 0.22 | 0.42 |
| AUD Diagnosis | 1.74 [0.60, 5.09] | 0.31 | 0.49 |
| **Time to First Conduct Disorder Symptoms** | | | |
| Initiation | 2.31 [1.72, 3.09] | 2.00E-08 | 5.00E-07 |
| Intoxication | 1.12 [0.76, 1.66] | 0.57 | 0.68 |
| AUD Symptom | 1.63 [0.99, 2.68] | 0.053 | 0.13 |
| AUD Diagnosis | 0.65 [0.29, 1.46] | 0.30 | 0.49 |
| **Time to First Oppositional Defiant Disorder Symptoms** | | | |
| Initiation | 1.63 [0.99, 2.69] | 0.053 | 0.13 |
| Intoxication | 0.85 [0.44, 1.63] | 0.62 | 0.70 |
| AUD Symptom | 1.49 [0.67, 3.33] | 0.33 | 0.49 |
| AUD Diagnosis | 1.35 [0.55, 3.33] | 0.52 | 0.65 |
| **African-American ancestry** | | | |
| **Alcohol Milestone** | **HR [95% CI]** | **p** | **p_FDR_** |
| **Time to First Major Depressive Episode** | | | |
| Initiation | 1.19 [0.85, 1.67] | 0.31 | 0.62 |
| Intoxication | 0.85 [0.56, 1.29] | 0.45 | 0.77 |
| AUD Symptom | 0.88 [0.56, 1.39] | 0.59 | 0.78 |
| AUD Diagnosis | 1.66 [1.02, 2.69] | 0.040 | 0.45 |
| **Time to First Suicidal Ideation** | | | |
| Initiation | 1.51 [0.93, 2.45] | 0.096 | 0.46 |
| Intoxication | 0.70 [0.38, 1.29] | 0.25 | 0.55 |
| AUD Symptom | 1.25 [0.61, 2.54] | 0.54 | 0.78 |
| AUD Diagnosis | 2.62 [1.28, 5.36] | 8.10E-03 | 0.19 |
| **Time to First Panic Disorder Symptoms** ^b^ | | | |
| Initiation | 1.31 [0.71, 2.43] | 0.20 | 0.53 |
| Intoxication | 0.60 [0.19, 1.90] | 0.056 | 0.45 |
| AUD Symptom | 2.59 [0.78, 8.58] | 0.14 | 0.48 |
| AUD Diagnosis | 0.95 [0.10, 8.73] | 0.18 | 0.53 |
| **Time to First Social Anxiety Disorder Symptoms** ^b^ | | | |
| Initiation | 1.05 [0.26, 4.17] | 0.95 | 0.99 |
| Intoxication | 0.70 [0.13, 3.85] | 0.68 | 0.78 |
| AUD Symptom | 0.55 [0.06, 5.42] | 0.61 | 0.78 |
| AUD Diagnosis | 5.78E-09 [0, Inf] | 1.00 | 1.00 |
| **Time to First Conduct Disorder Symptoms** | | | |
| Initiation | 0.89 [0.54, 1.47] | 0.66 | 0.78 |
| Intoxication | 1.67 [0.72, 3.91] | 0.23 | 0.55 |
| AUD Symptom | 0.31 [0.08, 1.22] | 0.094 | 0.46 |
| AUD Diagnosis | 0.37 [0.02, 5.96] | 0.48 | 0.77 |
| **Time to First Oppositional Defiant Disorder Symptoms** ^b^ | | | |
| Initiation | 1.15 [0.61, 2.19] | 0.66 | 0.78 |
| Intoxication | 0.57 [0.18, 1.85] | 0.35 | 0.65 |
| AUD Symptom | 2.49 [0.75, 8.26] | 0.14 | 0.48 |
| AUD Diagnosis | 0.82 [0.09, 7.88] | 0.87 | 0.95 |

*Note.* Sxs = Symptoms. p_FDR_ reflects false discovery rate-corrected p-values across all 25 (European ancestry) and, separately, across all 24 (African-American ancestry) tests. ^a^ To address proportional hazards violations, symptoms were examined at discrete age periods. ^b^ These models did not converge, so results should be interpreted with caution.

**Supplementary Table 9.** Cox Proportional Hazard Model Results for Polygenic Scores and Alcohol Transitions

| **PGS** | **HR [95% CI]** | **p** | **p_FDR_** |
| --- | --- | --- | --- |
| **Time to First Drink** | | | |
| Major Depressive Disorder (EUR) | 0.99 [0.91, 1.07] | 0.83 | 0.83 |
| *Major Depressive Disorder (AFR)* | *0.95 [0.87, 1.04]* | *0.29* | --- |
| Generalized Anxiety Disorder (EUR) | 1.01 [0.94, 1.08] | 0.79 | 0.83 |
| Neuroticism (EUR) | 1.03 [0.96, 1.11] | 0.42 | 0.83 |
| ADHD (EUR) | 1.04 [0.97, 1.11] | 0.29 | 0.32 |
| Common Executive Function (EUR) | 0.96 [0.89, 1.02] | 0.20 | 0.40 |
| Risk Tolerance (EUR) | 1.06 [0.99, 1.14] | 0.087 | 0.19 |
| Drinks Per Week (EUR) | 1.11 [1.03, 1.19] | 3.60E-03 | --- |
| Problematic Alcohol Use (EUR) | 1.06 [0.99, 1.14] | 0.099 | --- |
| *Drinks Per Week (AFR)* | *0.96 [0.88, 1.06]* | *0.43* | *---* |
| *Problematic Alcohol Use (AFR)* | *1.15 [1.05, 1.26]* | *3.00E-03* | *---* |
| **First Drink to First Intoxication** | | | |
| Major Depressive Disorder (EUR) | 0.98 [0.92, 1.05] | 0.65 | 0.83 |
| *Major Depressive Disorder (AFR)* | *0.96 [0.87, 1.05]* | *0.33* | --- |
| Generalized Anxiety Disorder (EUR) | 0.99 [0.93, 1.05] | 0.72 | 0.83 |
| Neuroticism (EUR) | 1.02 [0.96, 1.09] | 0.57 | 0.83 |
| ADHD (EUR) | 0.95 [0.90, 1.01] | 0.087 | 0.19 |
| Common Executive Function (EUR) | 1.00 [0.95, 1.06] | 0.99 | 0.99 |
| Risk Tolerance (EUR) | 1.05 [0.99, 1.11] | 0.097 | 0.19 |
| Drinks Per Week (EUR) | 1.06 [1.00, 1.12] | 0.060 | --- |
| Problematic Alcohol Use (EUR) | 1.00 [0.95, 1.06] | 0.97 | --- |
| *Drinks Per Week (AFR)* | *1.05 [0.95, 1.16]* | *0.32* | *---* |
| *Problematic Alcohol Use (AFR)* | *0.94 [0.85, 1.03]* | *0.17* | *---* |
| **First Drink to First AUD Symptom** | | | |
| Major Depressive Disorder (EUR) | 0.97 [0.90, 1.05] | 0.43 | 0.83 |
| *Major Depressive Disorder (AFR)* | *1.00 [0.91, 1.11]* | *0.93* | --- |
| Generalized Anxiety Disorder (EUR) | 1.07 [1.00, 1.15] | 0.035 | 0.35 |
| Neuroticism (EUR) | 0.99 [0.92, 1.06] | 0.69 | 0.83 |
| ADHD (EUR) | 1.06 [0.99, 1.13] | 0.08 | 0.19 |
| Common Executive Function (EUR) | 1.02 [0.96, 1.08] | 0.54 | 0.72 |
| Risk Tolerance (EUR) | 0.98 [0.92, 1.04] | 0.47 | 0.54 |
| Drinks Per Week (EUR) | 1.15 [1.08, 1.23] | 3.20E-05 | --- |
| Problematic Alcohol Use (EUR) | 0.98 [0.92, 1.05] | 0.58 | --- |
| *Drinks Per Week (AFR)* | *1.09 [0.98, 1.22]* | *0.12* | *---* |
| *Problematic Alcohol Use (AFR)* | *1.01 [0.90, 1.12]* | *0.93* | *---* |
| **First Drink to First AUD Diagnosis** | | | |
| Major Depressive Disorder (EUR) | 0.96 [0.88, 1.05] | 0.35 | 0.83 |
| *Major Depressive Disorder (AFR)* | *1.00 [0.88, 1.13]* | *0.94* | --- |
| Generalized Anxiety Disorder (EUR) | 1.08 [1.00, 1.16] | 0.059 | 0.35 |
| Neuroticism (EUR) | 1.06 [0.98, 1.16] | 0.16 | 0.64 |
| ADHD (EUR) | 1.03 [0.95, 1.11] | 0.45 | 0.54 |
| Common Executive Function (EUR) | 1.06 [0.98, 1.14] | 0.14 | 0.40 |
| Risk Tolerance (EUR) | 0.98 [0.91, 1.05] | 0.54 | 0.42 |
| Drinks Per Week (EUR) | 1.12 [1.04, 1.21] | 4.20E-03 | *---* |
| Problematic Alcohol Use (EUR) | 1.00 [0.93, 1.08] | 0.95 | *---* |
| *Drinks Per Week (AFR)* | *0.99 [0.87, 1.13]* | *0.85* | *---* |
| *Problematic Alcohol Use (AFR)* | *1.07 [0.94, 1.23]* | *0.32* | *---* |

*Note.* EUR = PCA-selected European ancestry; AFR = PCA-selected African-American ancestry. p_FDR_ reflects false discovery rate-corrected p-values across all 12 tests for internalizing, all 8 tests for externalizing, and 4 tests for executive function in the EUR ancestry subsample.

**Supplementary Table 10.** Cox Proportional Hazard Model Results for Polygenic Scores and Transitions from First Intoxication

| **PGS** | **HR [95% CI]** | **p** | **p_FDR_** |
| --- | --- | --- | --- |
| **First Intoxication to First AUD Symptom** | | | |
| Major Depressive Disorder (EUR) | 0.95 [0.88, 1.02] | 0.15 | 0.20 |
| *Major Depressive Disorder (AFR)* | *1.00 [0.88, 1.13]* | *0.94* | --- |
| Generalized Anxiety Disorder (EUR) | 1.10 [1.03, 1.18] | 4.10E-03 | 0.025 |
| Neuroticism (EUR) | 1.00 [0.93, 1.08] | 0.95 | 0.95 |
| ADHD (EUR) | 1.07 [1.00, 1.14] | 0.041 | 0.082 |
| Common Executive Function (EUR) | 1.05 [0.98, 1.11] | 0.16 | 0.15 |
| Risk Tolerance (EUR) | 0.93 [0.87, 0.99] | 0.033 | 0.082 |
| Drinks Per Week (EUR) | 1.14 [1.06, 1.22] | 1.90E-04 | *---* |
| Problematic Alcohol Use (EUR) | 1.00 [0.93, 1.07] | 0.93 | *---* |
| *Drinks Per Week (AFR)* | *1.04 [0.94, 1.16]* | *0.44* | *---* |
| *Problematic Alcohol Use (AFR)* | *1.06 [0.94 ,1.19]* | *0.35* | *---* |
| **First Intoxication to First AUD Diagnosis** | | | |
| Major Depressive Disorder (EUR) | 0.94 [0.87, 1.02] | 0.16 | 0.20 |
| *Major Depressive Disorder (AFR)* | *1.00 [0.88, 1.13]* | *0.99* | --- |
| Generalized Anxiety Disorder (EUR) | 1.09 [1.02, 1.18] | 0.018 | 0.054 |
| Neuroticism (EUR) | 1.06 [0.98, 1.15] | 0.17 | 0.20 |
| ADHD (EUR) | 1.05 [0.98, 1.13] | 0.20 | 0.20 |
| Common Executive Function (EUR) | 1.08 [1.01, 1.16] | 0.025 | 0.050 |
| Risk Tolerance (EUR) | 0.95 [0.89, 1.02] | 0.19 | 0.20 |
| Drinks Per Week (EUR) | 1.09 [1.01, 1.18] | 0.022 | *---* |
| Problematic Alcohol Use (EUR) | 1.01 [0.94, 1.09] | 0.78 | *---* |
| *Drinks Per Week (AFR)* | *1.00 [0.88, 1.14]* | *0.96* | *---* |
| *Problematic Alcohol Use (AFR)* | *1.13 [0.98, 1.31]* | *0.094* | *---* |

*Note.* EUR = PCA-selected European ancestry; AFR = PCA-selected African-American ancestry. p_FDR_ reflects false discovery rate-corrected p-values across all 6 tests for internalizing, all 4 tests for externalizing, and 2 tests for executive function in the EUR ancestry subsample.

**Supplementary Table 11.**  Post-hoc Results: PGS and Time from First Drink to First Mild, Moderate, and Severe AUD

| **PGS** | **HR [95% CI]** | **p** | **p_FDR_** |
| --- | --- | --- | --- |
| **Time from First Drink to First Mild AUD Diagnosis** | | | |
| Major Depressive Disorder (EUR) | 0.90 [0.82, 0.99] | .034 | 0.15 |
| *Major Depressive Disorder (AFR)* | *1.02 [0.88, 1.15]* | *0.88* | *---* |
| Generalized Anxiety Disorder (EUR) | 1.00 [0.92, 1.09] | 0.99 | 0.99 |
| Neuroticism (EUR) | 1.09 [1.00, 1.20] | 0.058 | 0.17 |
| ADHD (EUR) | 1.03 [0.95, 1.11] | 0.55 | 0.61 |
| Common Executive Function (EUR) | 1.09 [1.01, 1.18] | 0.026 | 0.039 |
| Risk Tolerance (EUR) | 1.03 [0.95, 1.12] | 0.45 | 0.61 |
| Drinks Per Week (EUR) | 1.10 [1.01, 1.20] | 0.022 | *---* |
| Problematic Alcohol Use (EUR) | 0.95 [0.87, 1.03] | 0.22 | *---* |
| *Drinks Per Week (AFR)* | 0.93 [0.81, 1.18] | 0.28 | *---* |
| *Problematic Alcohol Use (AFR)* | 1.02 [0.88, 1.15] | 0.81 | *---* |
| **Time from First Drink to First Moderate AUD Diagnosis** | | | |
| Major Depressive Disorder (EUR) | 1.00 [0.88, 1.15] | 0.95 | 0.99 |
| *Major Depressive Disorder (AFR)* | *0.82 [0.66, 1.03]* | *0.092* | --- |
| Generalized Anxiety Disorder (EUR) | 1.00 [0.89, 1.12] | 0.96 | 0.99 |
| Neuroticism (EUR) | 1.06 [0.93, 1.21] | 0.39 | 0.70 |
| ADHD (EUR) | 1.08 [0.96, 1.21] | 0.20 | 0.60 |
| Common Executive Function (EUR) | 1.08 [0.97, 1.21] | 0.15 | 0.15 |
| Risk Tolerance (EUR) | 0.97 [0.86, 1.09] | 0.61 | 0.56 |
| Drinks Per Week (EUR) | 1.13 [1.00, 1.27] | 0.041 | *---* |
| Problematic Alcohol Use (EUR) | 1.01 [0.90, 1.14] | 0.84 | *---* |
| *Drinks Per Week (AFR)* | 1.04 [0.84, 1.31] | 0.70 | *---* |
| *Problematic Alcohol Use (AFR)* | 1.24 [0.97, 1.59] | 0.092 | *---* |
| **Time from First Drink to First Severe AUD Diagnosis** | | | |
| Major Depressive Disorder (EUR) | 1.10 [0.92, 1.32] | 0.29 | 0.63 |
| *Major Depressive Disorder (AFR)* | *1.03 [0.78, 1.36]* | *0.83* | --- |
| Generalized Anxiety Disorder (EUR) | 1.25 [1.06, 1.46] | 6.60E-03 | 0.059 |
| Neuroticism (EUR) | 0.99 [0.83, 1.18] | 0.93 | 0.99 |
| ADHD (EUR) | 1.08 [0.92, 1.26] | 0.34 | 0.61 |
| Common Executive Function (EUR) | 0.85 [0.73, 0.98] | 0.039 | 0.045 |
| Risk Tolerance (EUR) | 0.91 [0.78, 1.05] | 0.20 | 0.60 |
| Drinks Per Week (EUR) | 0.98 [0.84, 1.15] | 0.84 | *---* |
| Problematic Alcohol Use (EUR) | 1.04 [0.89, 1.22] | 0.60 | *---* |
| *Drinks Per Week (AFR)* | 1.21 [0.91, 1.59] | 0.18 | *---* |
| *Problematic Alcohol Use (AFR)* | 1.06 [0.77, 1.45] | 0.72 | *---* |

*Note.* EUR = PCA-selected European ancestry; AFR = PCA-selected African-American ancestry. p_FDR_ reflects false discovery rate-corrected p-values across all 9 tests for internalizing, all 6 tests for externalizing, and 3 tests for executive function in the EUR ancestry subsample.

**Supplementary Table 12.** Cox Proportional Hazard Model Results for Polygenic Scores and Stage-Based Symptom Onsets

| **PGS** | **HR [95% CI]** | **p** | **p_FDR_** | |
| --- | --- | --- | --- | --- |
| **Time to First Major Depressive Episode** | | | | |
| Drinks Per Week (EUR) | 1.03 [0.95, 1.11] | 0.47 | 0.71 | |
| Problematic Alcohol Use (EUR) | 1.03 [0.95, 1.11] | 0.54 | 0.72 | |
| *Drinks Per Week (AFR)* | *0.99 [0.89, 1.09]* | *0.79* | --- | |
| *Problematic Alcohol Use (AFR)* | *1.00 [0.90, 1.11]* | *0.99* | --- | |
| **Time to First Suicidal Ideation** | | | | |
| Drinks Per Week (EUR) | 0.92 [0.83, 1.01] | 0.082 | 0.25 | |
| Problematic Alcohol Use (EUR) | 1.20 [1.08, 1.33] | 9.10E-04 | 0.011 | |
| *Drinks Per Week (AFR)* | *1.13 [0.98, 1.31]* | *0.10* | --- | |
| *Problematic Alcohol Use (AFR)* | *1.15 [0.98, 1.3]* | *0.081* | --- | |
| **Time to First Panic Disorder Symptoms** ^a^ | | | | |
| Drinks Per Week (EUR) | 1.01 [0.88, 1.15] | 0.92 | | 0.92 |
| Problematic Alcohol Use (EUR) | 1.03 [0.90, 1.18] | 0.68 | | 0.74 |
| *Drinks Per Week (AFR)* | *1.18 [0.88, 1.58]* | *0.28* | | --- |
| *Problematic Alcohol Use (AFR)* | *0.94 [0.70, 1.26]* | *0.66* | | --- |
| **Time to First Social Anxiety Disorder Symptoms** ^a^ | | | | |
| Drinks Per Week (EUR) | 0.92 [0.75, 1.11] | 0.37 | | 0.71 |
| Problematic Alcohol Use (EUR) | 1.08 [0.89, 1.31] | 0.45 | | 0.71 |
| *Drinks Per Week (AFR)* | *1.05 [0.75, 1.48]* | *0.77* | | --- |
| *Problematic Alcohol Use (AFR)* | *1.29 [0.88, 1.90]* | *0.20* | | --- |
| **Time to First Conduct Disorder Symptoms** | | | | |
| Drinks Per Week (EUR) | 1.13 [1.02, 1.25] | 0.023 | | 0.14 |
| Problematic Alcohol Use (EUR) | 1.12 [1.00, 1.24] | 0.041 | | 0.16 |
| *Drinks Per Week (AFR)* | *1.01 [0.90, 1.14]* | *0.85* | | --- |
| *Problematic Alcohol Use (AFR)* | *1.03 [0.90, 1.16]* | *0.70* | | --- |
| **Time to First Oppositional Defiant Disorder Symptoms** ^a^ | | | | |
| Drinks Per Week (EUR) | 0.97 [0.85, 1.10] | 0.64 | | 0.74 |
| Problematic Alcohol Use (EUR) | 1.07 [0.93, 1.22] | 0.35 | | 0.71 |
| *Drinks Per Week (AFR)* | *1.05 [0.90, 1.22]* | *0.54* | | --- |
| *Problematic Alcohol Use (AFR)* | *0.91 [0.78, 1.07]* | *0.27* | | --- |

*Note.* EUR = PCA-selected European ancestry; AFR = PCA-selected African-American ancestry. p_FDR_ reflects false discovery rate-corrected p-values across all 12 tests in EUR ancestry subsample. ^a^ Note that in the African-American ancestry subsample, these models did not converge, so estimates should be interpreted with caution.

**Supplementary Table 13.** Omnibus tests of effects of alcohol PGS and alcohol PGS*age interactions in EF trajectories

| **EF phenotype** | **Model** | **npar** | **BIC** | **Chisq** | **Df** | **p** | **p_FDR_** |
| --- | --- | --- | --- | --- | --- | --- | --- |
| ToL Excess Moves | Cov + age | 48 | 22161 |  |  |  |  |
|  | Cov + age + PGS | 50 | 22177 | 1.54 | 2 | 0.463 | --- |
|  | Cov + age*PGS | 56 | 22224 | 5.86 | 6 | 0.439 | --- |
| ToL Average Pickup Time | Cov + age | 48 | 114.98 |  |  |  |  |
|  | Cov + age + PGS | 50 | 131.75 | 0.717 | 2 | 0.699 | --- |
|  | Cov + age*PGS | 56 | 178.94 | 5.27 | 6 | 0.511 | --- |
| ToL Average Total Time | Cov + age | 48 | 185.91 |  |  |  |  |
|  | Cov + age + PGS | 50 | 202.60 | 0.792 | 2 | 0.673 | --- |
|  | Cov + age*PGS | 56 | 249.53 | 5.53 | 6 | 0.477 | --- |
| VST Forward Span | Cov + age | 48 | 20502 |  |  |  |  |
|  | Cov + age + PGS | 50 | 20518 | 0.438 | 2 | 0.803 | --- |
|  | Cov + age*PGS | 56 | 20569 | 2.42 | 6 | 0.878 | --- |
| VST Forward Total Correct | Cov + age | 48 | 27473 |  |  |  |  |
|  | Cov + age + PGS | 50 | 27490 | 0.0936 | 2 | 0.954 | --- |
|  | Cov + age*PGS | 56 | 27542 | 1.40 | 6 | 0.966 | --- |
| VST Backward Span | Cov + age | 48 | 17654 |  |  |  |  |
|  | Cov + age + PGS | 50 | 17670 | 1.33 | 2 | 0.515 | --- |
|  | Cov + age*PGS | 56 | 17714 | 7.89 | 6 | 0.247 | --- |
| VST Backward Total Correct | Cov + age | 48 | 24183 |  |  |  |  |
|  | Cov + age + PGS | 50 | 24199 | 1.21 | 2 | 0.545 | --- |
|  | Cov + age*PGS | 56 | 24241 | 10.46 | 6 | 0.107 | --- |

*Note.* ToL = Tower of London Test. VST = Visual Span Test. Cov = covariates, including first 10 ancestral principal components and non-alcohol PGS (i.e., MDD, GAD, NEUR, ADHD, RISKT, and cEF). PGS are DPW and PAU among individuals of PCA-selected European ancestry. Npar = number of parameters in the model. Df = degrees of freedom. P-values are for model comparisons between the model and the previous model, conducted via the anova() function in R.

**Supplementary Table 14.** Power analyses for alcohol transitions.

| **Phenotype** | **Initiation** | | **Intoxication** | | **AUD Symptom** | | **AUD Diagnosis** | |
| --- | --- | --- | --- | --- | --- | --- | --- | --- |
|  | **n** | **HR** | **n** | **HR** | **n** | **HR** | **n** | **HR** |
| MDE | 869 | 1.138 | 1,039 | 1.158 | 1,186 | 1.252 | 1,353 | 1.474 |
| Suicidal Ideation | 541 | 1.156 | 645 | 1.184 | 756 | 1.292 | 841 | 1.585 |
| Panic | 147 | 1.263 | 172 | 1.321 | 210 | 1.479 | 267 | 1.880 |
| SAD | 148 | 1.262 | 152 | 1.341 | 160 | 1.547 | 170 | 2.087 |
| Agoraphobia | 91 | 1.328 | 102 | 1.411 | 116 | 1.631 | 128 | 2.219 |
| ADHD Inatt | 1,464 | 1.140 | 1,284 | 1.153 | 1,288 | 1.269 | 1,303 | 1.680 |
| ADHD Hyper | 1,496 | 1.141 | 1,325 | 1.152 | 1,324 | 1.272 | 1,340 | 2.773 |
| Conduct | 737 | 1.145 | 910 | 1.168 | 983 | 1.289 | 1,040 | 1.747 |
| ODD | 482 | 1.163 | 532 | 1.197 | 551 | 1.330 | 576 | 1.732 |

*Note.* n represents the # of individuals endorsing each stage-based phenotype prior to age of onset of alcohol milestone or censoring. HR=hazard ratio required for 80% power. AUD=Alcohol Use Disorder. MDD=Major Depressive Episode. SAD=Social Anxiety Disorder. ODD=Oppositional Defiant Disorder. ADHD Inatt=ADHD Inattentive Symptoms. ADHD Hyper=ADHD Hyperactive Symptoms.

**Supplementary Table 15.** Power analyses for stage-based symptom onsets.

| **Phenotype** | **Initiation** | | **Intoxication** | | **AUD Symptom** | | **AUD Diagnosis** | |
| --- | --- | --- | --- | --- | --- | --- | --- | --- |
|  | **n** | **HR** | **n** | **HR** | **n** | **HR** | **n** | **HR** |
| MDE | 2,184 | 1.186 | 1,805 | 1.165 | 1,238 | 1.160 | 766 | 1.176 |
| Suicidal Ideation | 2,512 | 1.255 | 2,157 | 1.217 | 1,512 | 1.197 | 972 | 1.210 |
| Panic | 2,931 | 1.645 | 2,622 | 1.502 | 1,943 | 1.383 | 1,302 | 1.355 |
| SAD | 2,945 | 1.701 | 2,638 | 1.559 | 1,971 | 1.546 | 1,343 | 1.459 |
| Agoraphobia | 2,991 | 1.935 | 2,694 | 1.735 | 2,022 | 1.570 | 1,378 | 1.536 |
| Conduct | 2,628 | 1.315 | 2,265 | 1.259 | 1,572 | 1.232 | 988 | 1.248 |
| ODD | 2,579 | 1.289 | 2,280 | 1.257 | 1,652 | 1.242 | 1,082 | 1.257 |

*Note.* n represents the # of individuals endorsing each alcohol milestone prior to age of onset of stage-based phenotypes or censoring. HR=hazard ratio required for 80% power. AUD=Alcohol Use Disorder. MDD = Major Depressive Episode. SAD=Social Anxiety Disorder. ODD=Oppositional Defiant Disorder. ADHD symptoms are not depicted here due to extremely low endorsement following alcohol milestones (i.e., <10 individuals).

**Supplementary Figure 1.** Associations between internalizing, externalizing, and hazards of mild, moderate, and severe AUD


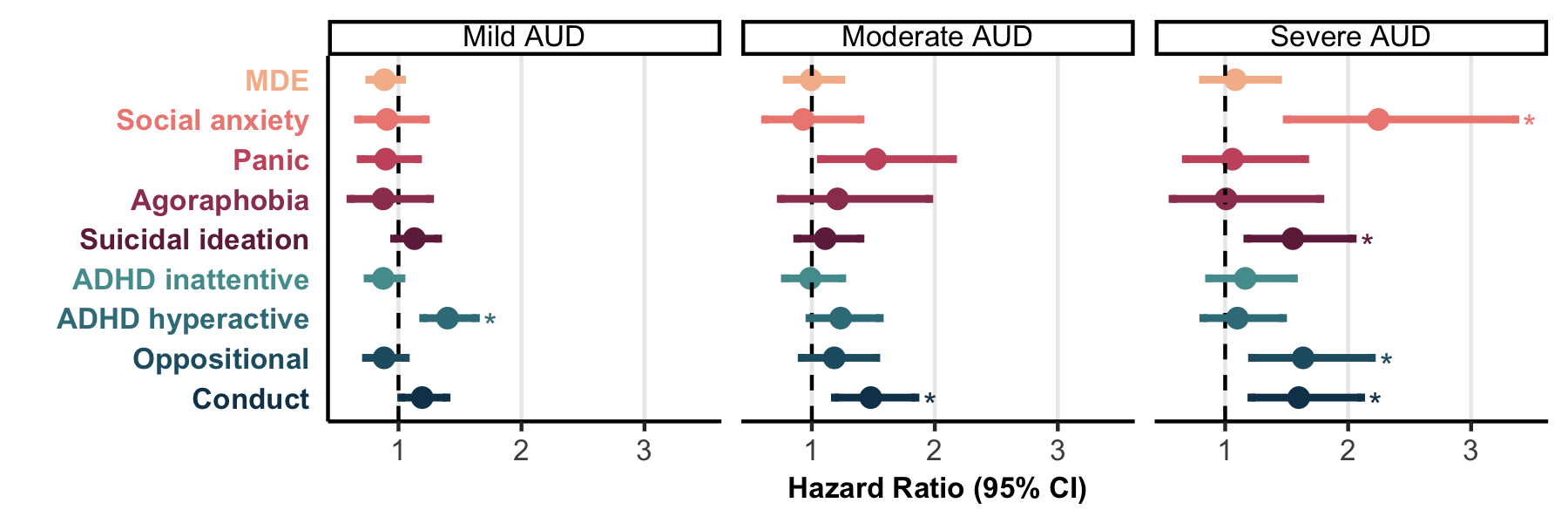


Associations between internalizing and externalizing phenotypes and hazards of progression from first drink to first mild, moderate, and severe alcohol use disorder (AUD). Point estimates reflect hazard ratios, and error bars represent 95% confidence intervals. Asterisks reflect estimates that survived FDR correction for all 15 tests of internalizing phenotypes and, separately, all 12 tests of externalizing phenotypes. All predictors were entered into the models simultaneously, alongside covariates.

**Supplementary Figure 2.** Associations between internalizing, externalizing, and executive function PGS and hazards of mild, moderate, and severe AUD


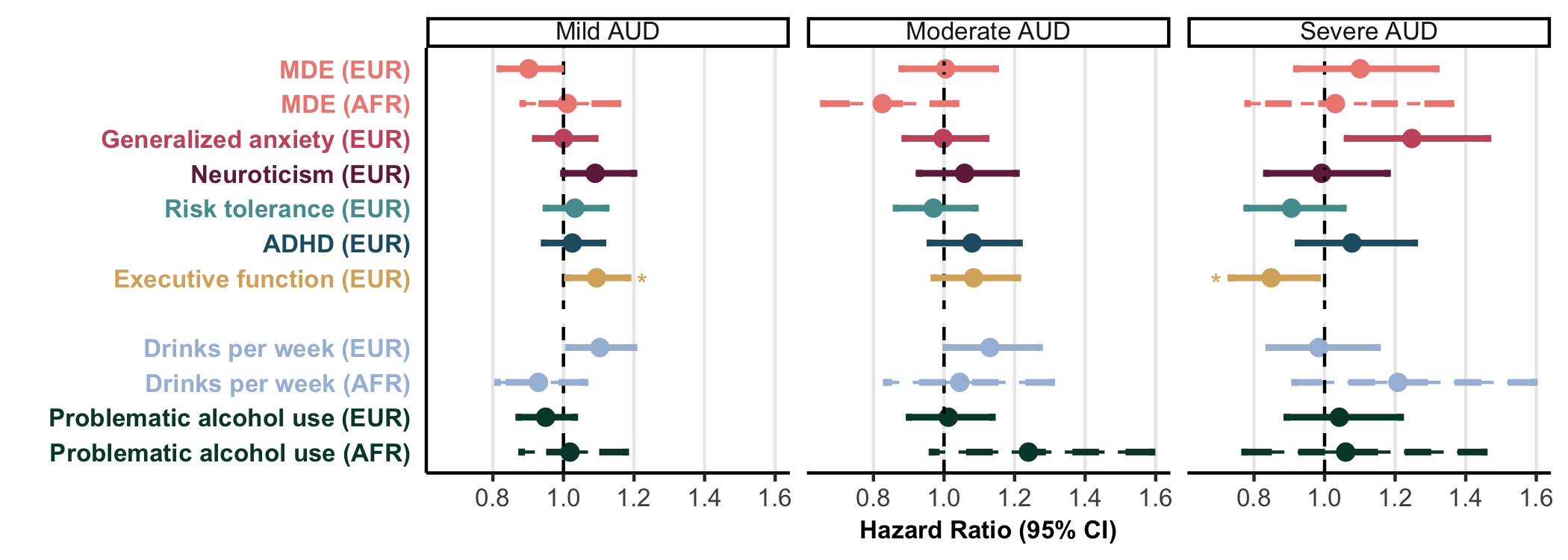


Associations between stage-based PGS and hazards of progression from first drink to first mild, moderate, and severe AUD. EUR = PCA-selected European ancestry; AFR = PCA-selected African ancestry. Point estimates reflect hazard ratios, and error bars represent 95% confidence intervals. AFR ancestry PGS associations are depicted with dotted lines. Alcohol PGS are included for comparison at the bottom of each plot. Asterisks reflect estimates that survived FDR correction, separately, for all 9 internalizing associations, all 6 externalizing associations, and all 3 executive function associations.

**Supplemental References**

*Dissertation Abstracts International, 34*(2-B), 891.

[Auton, A., Abecasis, G. R., Altshuler, D. M., Durbin, R. M., Abecasis, G. R., Bentley, D. R., …National Eye Institute, N. (2015). A global reference for human genetic variation. *Nature*, *526*(7571), Article 7571. https://doi.org/10.1038/nature15393](Auton,%20A.,%20Abecasis,%20G.%20R.,%20Altshuler,%20D.%20M.,%20Durbin,%20R.%20M.,%20Abecasis,%20G.%20R.,%20Bentley,%20D.%20R.,%20…National%20Eye%20Institute,%20N.%20(2015).%20A%20global%20reference%20for%20human%20genetic%20variation.%20Nature,%20526(7571),%20Article%207571.%20https://doi.org/10.1038/nature15393)

[Chang, C. C., Chow, C. C., Tellier, L. C., Vattikuti, S., Purcell, S. M., & Lee, J. J. (2015). Second-generation PLINK: Rising to the challenge of larger and richer datasets. *GigaScience*, *4*, 7. https://doi.org/10.1186/s13742-015-0047-8](https://www.zotero.org/google-docs/?FDBPEU)

[Corsi, P. M. (1972). Human memory and the medial temporal region of the brain. *Dissertation Abstracts International*, *34*(2-B), 891.](https://www.zotero.org/google-docs/?FDBPEU)

[Das, S., Forer, L., Schönherr, S., Sidore, C., Locke, A. E., Kwong, A., …Fuchsberger, C. (2016). Next-generation genotype imputation service and methods. *Nature Genetics*, *48*(10), Article 10. https://doi.org/10.1038/ng.3656](Das,%20S.,%20Forer,%20L.,%20Schönherr,%20S.,%20Sidore,%20C.,%20Locke,%20A.%20E.,%20Kwong,%20A.,%20…Fuchsberger,%20C.%20(2016).%20Next-generation%20genotype%20imputation%20service%20and%20methods.%20Nature%20Genetics,%2048(10),%20Article%2010.%20https://doi.org/10.1038/ng.3656)

[Davis, H., & Keller, F. (2002). *Colorado Assessment Tests (CATs), Version 1.2* (1.2) [Computer software].](https://www.zotero.org/google-docs/?FDBPEU)

[Delaneau, O., Zagury, J.-F., & Marchini, J. (2013). Improved whole-chromosome phasing for disease and population genetic studies. *Nature Methods*, *10*(1), 5–6. https://doi.org/10.1038/nmeth.2307](https://www.zotero.org/google-docs/?FDBPEU)

[Demontis, D., Walters, R. K., Martin, J., Mattheisen, M., Als, T. D., Agerbo, E., …Neale, B. M. (2019). Discovery of the first genome-wide significant risk loci for attention deficit/hyperactivity disorder. *Nature Genetics*, *51*(1), Article 1. https://doi.org/10.1038/s41588-018-0269-7](Demontis,%20D.,%20Walters,%20R.%20K.,%20Martin,%20J.,%20Mattheisen,%20M.,%20Als,%20T.%20D.,%20Agerbo,%20E.,%20…Neale,%20B.%20M.%20(2019).%20Discovery%20of%20the%20first%20genome-wide%20significant%20risk%20loci%20for%20attention%20deficit/hyperactivity%20disorder.%20Nature%20Genetics,%2051(1),%20Article%201.%20https://doi.org/10.1038/s41588-018-0269-7)

[Ge, T., Chen, C. Y., Ni, Y., Feng, Y. C. A., & Smoller, J. W. (2019). Polygenic prediction via Bayesian regression and continuous shrinkage priors. *Nat Commun*, *10*(1). https://doi.org/10.1038/s41467-019-09718-5](https://www.zotero.org/google-docs/?FDBPEU)

[Hatoum, A. S., Morrison, C. L., Mitchell, E. C., Lam, M., Benca-Bachman, C. E., Reineberg, A. E., …Friedman, N. P. (2023). Genome-wide Association Study Shows That Executive Functioning Is Influenced by GABAergic Processes and Is a Neurocognitive Genetic Correlate of Psychiatric Disorders. *Biological Psychiatry*, *93*(1), 59–70. https://doi.org/10.1016/j.biopsych.2022.06.034](Hatoum,%20A.%20S.,%20Morrison,%20C.%20L.,%20Mitchell,%20E.%20C.,%20Lam,%20M.,%20Benca-Bachman,%20C.%20E.,%20Reineberg,%20A.%20E.,%20…Friedman,%20N.%20P.%20(2023).%20Genome-wide%20Association%20Study%20Shows%20That%20Executive%20Functioning%20Is%20Influenced%20by%20GABAergic%20Processes%20and%20Is%20a%20Neurocognitive%20Genetic%20Correlate%20of%20Psychiatric%20Disorders.%20Biological%20Psychiatry,%2093(1),%2059–70.%20https://doi.org/10.1016/j.biopsych.2022.06.034)

[Hothorn, T., Bretz, F., Westfall, P., Heiberger, R. M., Schuetzenmeister, A., & Scheibe, S. (2023). *multcomp: Simultaneous Inference in General Parametric Models* (1.4-25) [Computer software]. https://cran.r-project.org/web/packages/multcomp/index.html](https://www.zotero.org/google-docs/?FDBPEU)

Howard, D. M., Adams, M. J., Clarke, T.-K., Hafferty, J. D., Gibson, J., Shirali, M., …McIntosh, A. M. (2019). Genome-wide meta-analysis of depression identifies 102 independent variants and highlights the importance of the prefrontal brain regions. *Nature Neuroscience*, *22*(3), Article 3. <https://doi.org/10.1038/s41593-018-0326-7>

[Kamarajan, C., Ardekani, B. A., Pandey, A. K., Kinreich, S., Pandey, G., Chorlian, D. B., …Porjesz, B. (2020). Random Forest Classification of Alcohol Use Disorder Using fMRI Functional Connectivity, Neuropsychological Functioning, and Impulsivity Measures. *Brain Sciences*, *10*(2), 115. https://doi.org/10.3390/brainsci10020115](Kamarajan,%20C.,%20Ardekani,%20B.%20A.,%20Pandey,%20A.%20K.,%20Kinreich,%20S.,%20Pandey,%20G.,%20Chorlian,%20D.%20B.,%20…Porjesz,%20B.%20(2020).%20Random%20Forest%20Classification%20of%20Alcohol%20Use%20Disorder%20Using%20fMRI%20Functional%20Connectivity,%20Neuropsychological%20Functioning,%20and%20Impulsivity%20Measures.%20Brain%20Sciences,%2010(2),%20115.%20https://doi.org/10.3390/brainsci10020115)

[Karlsson Linnér, R., Biroli, P., Kong, E., Meddens, S. F. W., Wedow, R., Fontana, M. A., …Beauchamp, J. P. (2019). Genome-wide association analyses of risk tolerance and risky behaviors in over 1 million individuals identify hundreds of loci and shared genetic influences. *Nature Genetics*, *51*(2), 245–257. https://doi.org/10.1038/s41588-018-0309-3](file:////Users/sarahpaul/Documents/Paperpreparation/COGA%20stage-based/Karlsson%20Linnér,%20R.,%20Biroli,%20P.,%20Kong,%20E.,%20Meddens,%20S.%20F.%20W.,%20Wedow,%20R.,%20Fontana,%20M.%20A.,%20…Beauchamp,%20J.%20P.%20(2019).%20Genome-wide%20association%20analyses%20of%20risk%20tolerance%20and%20risky%20behaviors%20in%20over%201%20million%20individuals%20identify%20hundreds%20of%20loci%20and%20shared%20genetic%20influences.%20Nature%20Genetics,%2051(2),%20245–257.%20https:/doi.org/10.1038/s41588-018-0309-3)

Kim, J. H. (2019). Multicollinearity and misleading statistical results. *Korean Journal of Anesthesiology*, *72*(6), 558–569. https://doi.org/10.4097/kja.19087

[Kranzler, H. R., Zhou, H., Kember, R. L., Vickers Smith, R., Justice, A. C., Damrauer, S., …Gelernter, J. (2019). Genome-wide association study of alcohol consumption and use disorder in 274,424 individuals from multiple populations. *Nature Communications*, *10*(1). https://doi.org/10.1038/s41467-019-09480-8](file:////Users/sarahpaul/Documents/Paperpreparation/COGA%20stage-based/Kranzler,%20H.%20R.,%20Zhou,%20H.,%20Kember,%20R.%20L.,%20Vickers%20Smith,%20R.,%20Justice,%20A.%20C.,%20Damrauer,%20S.,%20…Gelernter,%20J.%20(2019).%20Genome-wide%20association%20study%20of%20alcohol%20consumption%20and%20use%20disorder%20in%20274,424%20individuals%20from%20multiple%20populations.%20Nature%20Communications,%2010(1).%20https:/doi.org/10.1038/s41467-019-09480-8)

Kutner, M. H., Nachtsheim, C. J., Neter, J., & Li, W. (2005). *Applied linear statistical models* (5th ed). McGraw-Hill Irwin.

Levey, D. F., Gelernter, J., Polimanti, R., Zhou, H., Cheng, Z., Aslan, M., …Stein, M. B. (2020). Reproducible Genetic Risk Loci for Anxiety: Results From ∼200,000 Participants in the Million Veteran Program. *The American Journal of Psychiatry*, *177*(3), 223–232. <https://doi.org/10.1176/appi.ajp.2019.19030256>

[Levey, D. F., Stein, M. B., Wendt, F. R., Pathak, G. A., Zhou, H., Aslan, M., …Gelernter, J. (2021). Bi-ancestral depression GWAS in the Million Veteran Program and meta-analysis in >1.2 million individuals highlight new therapeutic directions. *Nature Neuroscience*, *24*(7), 954–963. https://doi.org/10.1038/s41593-021-00860-2](Levey,%20D.%20F.,%20Stein,%20M.%20B.,%20Wendt,%20F.%20R.,%20Pathak,%20G.%20A.,%20Zhou,%20H.,%20Aslan,%20M.,%20…Gelernter,%20J.%20(2021).%20Bi-ancestral%20depression%20GWAS%20in%20the%20Million%20Veteran%20Program%20and%20meta-analysis%20in%20%3e1.2%20million%20individuals%20highlight%20new%20therapeutic%20directions.%20Nature%20Neuroscience,%2024(7),%20954–963.%20https://doi.org/10.1038/s41593-021-00860-2)

[Mathias, R. A., Taub, M. A., Gignoux, C. R., Fu, W., Musharoff, S., O’Connor, T. D., …Barnes, K. C. (2016). A continuum of admixture in the Western Hemisphere revealed by the African Diaspora genome. *Nature Communications*, *7*, 12522. https://doi.org/10.1038/ncomms12522](Mathias,%20R.%20A.,%20Taub,%20M.%20A.,%20Gignoux,%20C.%20R.,%20Fu,%20W.,%20Musharoff,%20S.,%20O’Connor,%20T.%20D.,%20…Barnes,%20K.%20C.%20(2016).%20A%20continuum%20of%20admixture%20in%20the%20Western%20Hemisphere%20revealed%20by%20the%20African%20Diaspora%20genome.%20Nature%20Communications,%207,%2012522.%20https://doi.org/10.1038/ncomms12522)

[McCarthy, S., Das, S., Kretzschmar, W., Delaneau, O., Wood, A. R., Teumer, A., …the Haplotype Reference Consortium. (2016). A reference panel of 64,976 haplotypes for genotype imputation. *Nature Genetics*, *48*(10), Article 10. https://doi.org/10.1038/ng.3643](McCarthy,%20S.,%20Das,%20S.,%20Kretzschmar,%20W.,%20Delaneau,%20O.,%20Wood,%20A.%20R.,%20Teumer,%20A.,%20…the%20Haplotype%20Reference%20Consortium.%20(2016).%20A%20reference%20panel%20of%2064,976%20haplotypes%20for%20genotype%20imputation.%20Nature%20Genetics,%2048(10),%20Article%2010.%20https://doi.org/10.1038/ng.3643)

[Meyers, J. L., Chorlian, D. B., Johnson, E. C., Pandey, A. K., Kamarajan, C., Salvatore, J. E., …Porjesz, B. (2019). Association of Polygenic Liability for Alcohol Dependence and EEG Connectivity in Adolescence and Young Adulthood. *Brain Sciences*, *9*(10), 280. https://doi.org/10.3390/brainsci9100280](Meyers,%20J.%20L.,%20Chorlian,%20D.%20B.,%20Johnson,%20E.%20C.,%20Pandey,%20A.%20K.,%20Kamarajan,%20C.,%20Salvatore,%20J.%20E.,%20…Porjesz,%20B.%20(2019).%20Association%20of%20Polygenic%20Liability%20for%20Alcohol%20Dependence%20and%20EEG%20Connectivity%20in%20Adolescence%20and%20Young%20Adulthood.%20Brain%20Sciences,%209(10),%20280.%20https://doi.org/10.3390/brainsci9100280)

[Milner, B. (1971). Interhemispheric differences in the localization of psychological processes in man. *British Medical Bulletin*, *27*(3), 272–277. https://doi.org/10.1093/oxfordjournals.bmb.a070866](https://www.zotero.org/google-docs/?FDBPEU)

Nagel, M., Jansen, P. R., Stringer, S., Watanabe, K., de Leeuw, C. A., Bryois, J., …Posthuma, D. (2018). Meta-analysis of genome-wide association studies for neuroticism in 449,484 individuals identifies novel genetic loci and pathways. *Nature Genetics*, *50*(7), 920–927. <https://doi.org/10.1038/s41588-018-0151-7>

[O’Connell, J. R., & Weeks, D. E. (1998). PedCheck: A program for identification of genotype incompatibilities in linkage analysis. *American Journal of Human Genetics*, *63*(1), 259–266. https://doi.org/10.1086/301904](https://www.zotero.org/google-docs/?FDBPEU)

[Price, A. L., Patterson, N. J., Plenge, R. M., Weinblatt, M. E., Shadick, N. A., & Reich, D. (2006). Principal components analysis corrects for stratification in genome-wide association studies. *Nature Genetics*, *38*(8), Article 8. https://doi.org/10.1038/ng1847](https://www.zotero.org/google-docs/?FDBPEU)

[Purcell, S., Neale, B., Todd-Brown, K., Thomas, L., Ferreira, M. A. R., Bender, D., …Sham, P. C. (2007). PLINK: A Tool Set for Whole-Genome Association and Population-Based Linkage Analyses. *American Journal of Human Genetics*, *81*(3), 559–575.](https://www.zotero.org/google-docs/?FDBPEU)

[Qiu, W., Chavarro, J., Lazarus, R., Rosner, B., & Ma, J. (2021). *powerSurvEpi: Power and Sample Size Calculation for Survival Analysis of Epidemiological Studies* (0.1.3) [Computer software]. https://cran.r-project.org/web/packages/powerSurvEpi/index.html](https://www.zotero.org/google-docs/?FDBPEU)

[Ruan, Y., Lin, Y.-F., Feng, Y.-C. A., Chen, C.-Y., Lam, M., Guo, Z.,…Ge, T. (2022). Improving polygenic prediction in ancestrally diverse populations. *Nature Genetics*, *54*(5), Article 5. https://doi.org/10.1038/s41588-022-01054-7](Ruan,%20Y.,%20Lin,%20Y.-F.,%20Feng,%20Y.-C.%20A.,%20Chen,%20C.-Y.,%20Lam,%20M.,%20Guo,%20Z.,…Ge,%20T.%20(2022).%20Improving%20polygenic%20prediction%20in%20ancestrally%20diverse%20populations.%20Nature%20Genetics,%2054(5),%20Article%205.%20https://doi.org/10.1038/s41588-022-01054-7)

[Saunders, G. R. B., Wang, X., Chen, F., Jang, S.-K., Liu, M., Wang, C., …Vrieze, S. (2022). Genetic diversity fuels gene discovery for tobacco and alcohol use. *Nature*, *612*(7941), Article 7941. https://doi.org/10.1038/s41586-022-05477-4](Saunders,%20G.%20R.%20B.,%20Wang,%20X.,%20Chen,%20F.,%20Jang,%20S.-K.,%20Liu,%20M.,%20Wang,%20C.,%20…Vrieze,%20S.%20(2022).%20Genetic%20diversity%20fuels%20gene%20discovery%20for%20tobacco%20and%20alcohol%20use.%20Nature,%20612(7941),%20Article%207941.%20https://doi.org/10.1038/s41586-022-05477-4)

[Shallice, T. (1982). Specific impairments of planning. *Philosophical Transactions of the Royal Society of London. Series B, Biological Sciences*, *298*(1089), 199–209. https://doi.org/10.1098/rstb.1982.0082](https://www.zotero.org/google-docs/?FDBPEU)

[Subbie-Saenz de Viteri, S., Pandey, A., Pandey, G., Kamarajan, C., Smith, R., Anokhin, A., …Meyers, J. L. (2020). Pathways to post-traumatic stress disorder and alcohol dependence: Trauma, executive functioning, and family history of alcoholism in adolescents and young adults. *Brain and Behavior*, *10*(11), e01789. https://doi.org/10.1002/brb3.1789](Subbie-Saenz%20de%20Viteri,%20S.,%20Pandey,%20A.,%20Pandey,%20G.,%20Kamarajan,%20C.,%20Smith,%20R.,%20Anokhin,%20A.,%20…Meyers,%20J.%20L.%20(2020).%20Pathways%20to%20post-traumatic%20stress%20disorder%20and%20alcohol%20dependence:%20Trauma,%20executive%20functioning,%20and%20family%20history%20of%20alcoholism%20in%20adolescents%20and%20young%20adults.%20Brain%20and%20Behavior,%2010(11),%20e01789.%20https://doi.org/10.1002/brb3.1789)

[Zhou, H., Sealock, J. M., Sanchez-Roige, S., Clarke, T. K., Levey, D. F., Cheng, Z., Li, B., …Gelernter, J. (2020). Genome-wide meta-analysis of problematic alcohol use in 435,563 individuals yields insights into biology and relationships with other traits. *Nature Neuroscience*, *23*(7), 809–818. https://doi.org/10.1038/s41593-020-0643-5](Zhou,%20H.,%20Sealock,%20J.%20M.,%20Sanchez-Roige,%20S.,%20Clarke,%20T.%20K.,%20Levey,%20D.%20F.,%20Cheng,%20Z.,%20Li,%20B.,%20…Gelernter,%20J.%20(2020).%20Genome-wide%20meta-analysis%20of%20problematic%20alcohol%20use%20in%20435,563%20individuals%20yields%20insights%20into%20biology%20and%20relationships%20with%20other%20traits.%20Nature%20Neuroscience,%2023(7),%20809–818.%20https://doi.org/10.1038/s41593-020-0643-5)
